# Predict long-range enhancer regulation based on protein–protein interactions between transcription factors

**DOI:** 10.1093/nar/gkab841

**Published:** 2021-09-27

**Authors:** Hao Wang, Binbin Huang, Jianrong Wang

**Affiliations:** Department of Computational Mathematics, Science and Engineering, Michigan State University, 428 S. Shaw Ln., East Lansing, MI 48824, USA; Department of Computational Mathematics, Science and Engineering, Michigan State University, 428 S. Shaw Ln., East Lansing, MI 48824, USA; Department of Computational Mathematics, Science and Engineering, Michigan State University, 428 S. Shaw Ln., East Lansing, MI 48824, USA

## Abstract

Long-range regulation by distal enhancers plays critical roles in cell-type specific transcriptional programs. Computational predictions of genome-wide enhancer–promoter interactions are still challenging due to limited accuracy and the lack of knowledge on the molecular mechanisms. Based on recent biological investigations, the protein–protein interactions (PPIs) between transcription factors (TFs) have been found to participate in the regulation of chromatin loops. Therefore, we developed a novel predictive model for cell-type specific enhancer–promoter interactions by leveraging the information of TF PPI signatures. Evaluated by a series of rigorous performance comparisons, the new model achieves superior performance over other methods. The model also identifies specific TF PPIs that may mediate long-range regulatory interactions, revealing new mechanistic understandings of enhancer regulation. The prioritized TF PPIs are associated with genes in distinct biological pathways, and the predicted enhancer–promoter interactions are strongly enriched with cis-eQTLs. Most interestingly, the model discovers enhancer-mediated trans-regulatory links between TFs and genes, which are significantly enriched with trans-eQTLs. The new predictive model, along with the genome-wide analyses, provides a platform to systematically delineate the complex interplay among TFs, enhancers and genes in long-range regulation. The novel predictions also lead to mechanistic interpretations of eQTLs to decode the genetic associations with gene expression.

## INTRODUCTION

Cell-type specific transcriptional regulation plays important roles in differentiation and development (1–13). In addition to proximal regulatory elements, e.g. promoters, which are located around transcriptional start sites (TSS) of genes, distal enhancers provide complex and precise controls on gene expression through long-range regulation (14,15). Based on recent genome-wide enhancer annotations from ENCODE and Roadmap Epigenomics projects (16,17), hundreds of thousands of putative enhancers across the whole human genome have been identified, especially in non-coding regions, highlighting the biological impacts of enhancer regulation. Although a series of computational algorithms have been developed to predict the genomic locations of cell-type specific enhancers (18,19), it remains challenging to identify the specific target genes regulated by enhancers in different cell-types or tissues. Unlike promoters, enhancers are usually located far away from their target genes along the genome (20) and the nearest genes may not be regulated by a proximal enhancer (21). In three-dimensional (3D) space, an enhancer and its target genes are placed close to each other through long-range chromatin interactions, *i.e*. enhancer–promoter interactions (22).

The discoveries of tissue-specific long-range enhancer regulation have the potential to enable novel insights in a wide range of different biological studies. As one of the canonical examples, long-range regulation by distal enhancers play pivotal roles in controlling the tissue and condition-specific expression of the mouse $\beta$-globin (*Hbb*) gene expression (1,5,6). As another well-known example, the expression of the *Shh* gene in mouse limb bud is precisely regulated by a distal enhancer located 850 kb away, which is critical for the proper limb development (7–9,23). In addition to normal tissue development, the annotation of long-range enhancer regulation has also facilitated the interpretation of genetic variants underlying complex diseases. A non-coding genetic variant associated with obesity is located in an intron of the *FTO* gene but regulates the *IRX3* and *IRX5* genes that are located >400 kb away (2,10,24). Similar examples of long-range interactions linking disease-associated genetic variants to distal genes have also been found in studies of autoimmune diseases (3,4,11–13).

Given the functional importance of long-range enhancer regulation, experimental techniques have been developed to identify chromatin interactions linking distal enhancers to promoters of their target genes. Based on the pioneering chromosome conformation capture (3C) technology (25), along with its derivatives of 4C and 5C (26,27), the genome-wide version, i.e. Hi-C (28), has been applied to several human cell-types and tissues (16,29,30). Furthermore, the promoter-enriched genome conformation assay, Capture Hi-C (31), improves the resolution and cell-type specificity of the identified chromatin interactions for gene promoters (32). On the other hand, the method of chromatin interaction analysis with paired-end-tag sequencing (ChIA-PET) (33) was developed to capture long-range chromatin interactions associated with a protein of interest, such as a specific transcription factor (TF), with high-resolution and cell-type specificity (34). These cutting-edge technologies have generated large-scale chromatin contact maps for a number of cell-types or tissues in the human genome and other model species (16,29,30,34).

Although experimental techniques have substantially expanded the catalog of annotations for long-range chromatin interactions, there are several limitations that hinder in-depth analysis on cell-type specific enhancer–promoter interactions. First, the resolution of interacting genomic anchors profiled by Hi-C and Capture Hi-C is relatively low (∼5–10 kb genomic fragments) (29,31), which makes it difficult to pinpoint the specific enhancers involved in long-range regulation. Second, while Capture Hi-C and ChIA-PET experiments can discover cell-type or tissue-specific enhancer regulation, data generated by Hi-C experiments have been found to be largely invariant across different cell-types or tissues (35). Third, the background noise levels of Hi-C and Capture Hi-C datasets are high, leading to many false positive discoveries (36). Fourth, due to the dependency on specific protein antibodies, such as CTCF or RNA Pol II (34), each ChIA-PET experiment can only profile a subset of long-range interactions, resulting in large numbers of false negative interactions that are not identified (37).

Because of these limitations, computational models are needed to predict cell-type specific long-range enhancer regulation, based on integration of multi-omics signatures, e.g. genomics, transcriptomics, and epigenomics. Large-scale multi-omics data resources collected by the ENCODE and Roadmap Epigenomics projects contain the multi-view information of gene regulation (16), including gene expression, transcription factor binding and histone modifications. They can help to overcome the limitations of experimental techniques because they are cell-type or tissue specific (38), provide high-resolution signal landscape along the genome (39,40), have high signal-to-noise ratio (40), and cover the genomic binding sites for diverse transcription factors (16). The existing computational models of long-range enhancer–promoter interaction prediction can be grouped into two classes. For the first class, i.e. supervised algorithms, 3D chromatin interactions profiled by experimental techniques are used as labels for enhancer–promoter pairs. The commonly used features include: (i) cell-type specific gene expression based on RNA-seq data; (ii) enhancer activity based on specific epigenetic signals, such as H3K4me1, H3K27ac or DNase hypersensitivity; (iii) genomic separation distance between enhancers and gene promoters and (iv) correlations between gene expression and enhancer activity. Supervised methods incorporating some or all of these features include RIPPLE (41), FOCS (42), EAGLE (43) and JEME (44). As one of the most recently developed supervised methods, JEME (44) employs a combined approach of regression and random forest to predict long-range regulatory links between enhancers and genes. But it requires multi-omics datasets from a large panel of diverse cell-types and tissues as inputs, which is usually not available for users. The other two top-performing methods are IM-PET (45) and TargetFinder (46). These two algorithms not only integrate the features described above but also leverage additional features of transcription factor binding in promoters, enhancers, or genomic windows between enhancers and promoters. With respect to machine learning techniques, IM-PET employs a random forest model, and TargetFinder implements a boosting tree approach. For the second class, i.e. unsupervised algorithms, every enhancer–promoter pair is assigned with a score and then ranked based on the scores. Top-ranking enhancer–promoter pairs are predicted to interact with each other. The scores are generally based on genomic separation distance and co-activity patterns, e.g. correlations, between enhancers and genes (47–49). Based on a systematic performance evaluation analysis (50), supervised methods overall demonstrate better performance than unsupervised methods, but many of the supervised methods suffer from overfitting issues due to high model complexity (50) or excessively high-dimensional features that are often shared across training and testing sets (51). Furthermore, existing methods provide limited mechanistic insights on how specific long-range chromatin interactions are established to link distal enhancers with promoters of target genes (52).

**Figure 1.**
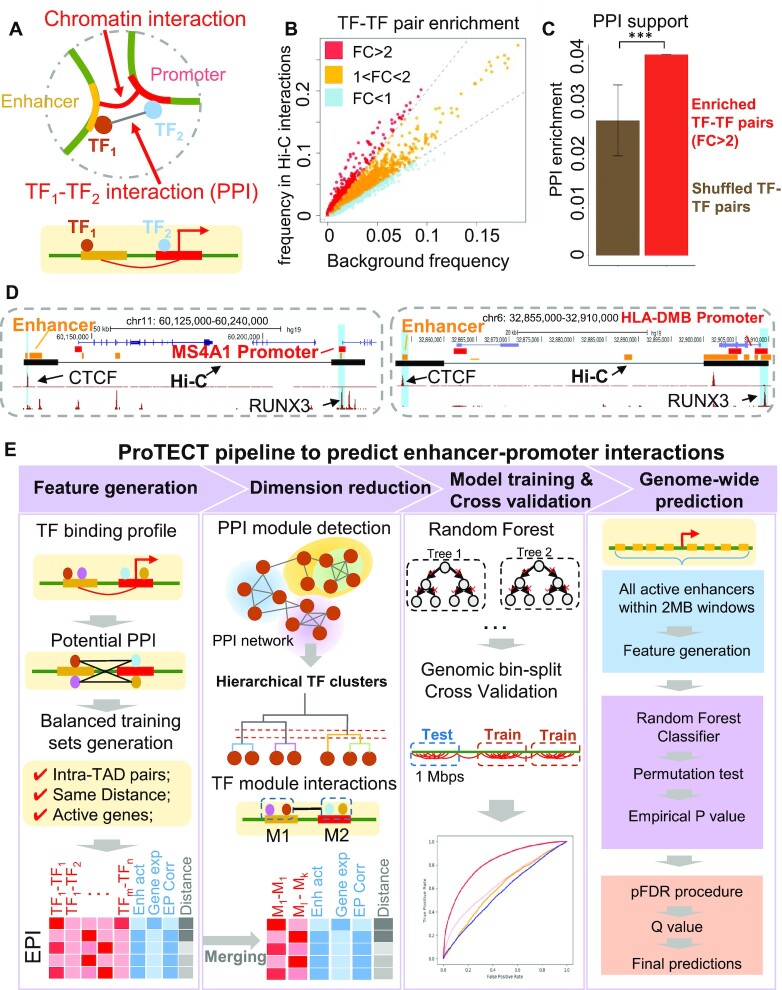
ProTECT infers long-range enhancer–promoter interactions based on TF PPI features. (**A**) The enhancer–promoter interactions are regulated by PPIs between enhancer-binding TFs (brown) and promoter-binding TFs (blue), which link distal enhancers (orange) to the proximity of promoters (red) in 3D chromatin structure. (**B**) Enrichment of TF–TF pairs in Hi-C interactions (y-axis) compared to background (x-axis). Points represent TF–TF pairs. Frequency is calculated as the fraction of enhancer-gene pairs containing the specific TF–TF pairs. Fold-change (FC) is the ratio of the frequency in Hi-C interactions over the frequency in background. TF–TF pairs are colored by the FC (red: FC > 2; orange: 1 < FC < 2; blue: FC < 1). (**C**) Enriched TF–TF pairs are supported by PPIs. The fraction of pairs supported by PPIs are calculated for the set of enriched TF–TF pairs (red). As controls, the TF members from the enriched TF–TF pairs are randomly paired (brown). Statistical test is done based on 1000 random repeats of controls (***P-value = 10^–3^). Error bar represents sd. (**D**) Examples of Hi-C interactions linking enhancers (orange) and promoters (red) showing enhancer-binding CTCF ChIP-seq peaks and promoter-binding RUNX3 ChIP-seq peaks in GM12878 cells. (**E**) The workflow of ProTECT algorithm. A balanced training dataset is generated with confounding factors controlled. A feature matrix summarizing cell-type specific TF PPI features, activity-based features (enhancer activity, gene expression, enhancer-gene activity correlation), and genomic distances is then constructed. A novel hierarchical network community detection-based approach is applied for feature dimension reduction. Based on the reduced feature matrix, a random forest model is trained, and rigorous genomic-bin split cross-validations are used for performance evaluations and comparisons. Using the trained predictive model, genome-wide high-confidence enhancer–promoter interactions are predicted based on stringent permutation statistical tests.

Interestingly, as shown by recent experimental studies (2,53–58), in addition to the binding of individual TFs on enhancers or promoters, the protein–protein interactions (PPIs) between TFs have been found to participate in the process of long-range chromatin interaction formation and thus, mediate distal enhancer to the proximity of target gene promoters (Figure 1A–D). For example, the PPI between the enhancer-binding and promoter-binding YY1s (i.e. YY1 dimerization) has been found to mediate enhancer–promoter contacts (59). The ChIA-PET data from mESCs suggests that the YY1–YY1 interactions largely participate in the connections between active enhancers and gene promoters (59). In a chromatin structure engineering study, based on a CRISPR-dCas9 system, two proteins (PYL1 and ABL1) are fused to dCas9 and are guided to bind on different genomic locations (60). Remarkably, the PYL1–ABL1 dimerization can establish novel long-range chromatin interactions, highlighting the mechanistic importance of PPIs in orchestrating chromatin loops. In addition, a couple of genome-wide analyses have also found that specific groups of transcription factors are enriched in cell-type specific long-range chromatin interactions (61–63). Within each group, some TF members can interact with each other and form protein complexes. As a representative example, a group of CTCF, RAD21, SMC3 and ZNF143 is found to be enriched in chromatin interactions (61), consistent with the chromatin loop extrusion model that CTCF and cohesin can interact with each other and regulate chromatin loops (64,65).

These observations strongly support the mechanistic hypothesis that specific TF PPIs, except intratypic dimerizations where TFs can only co-bind locally to DNA instead of across long-range distances, may mediate long-range enhancer regulation. Therefore, incorporation of TF PPIs as a new set of features into a machine learning model is expected to improve the accuracy of long-range enhancer–promoter interaction predictions. Moreover, the prioritized TF PPIs from the predictive model can further indicate the important transcription factors that facilitate long-range enhancer regulation, leading to novel understandings of enhancer biology. However, unlike basic enrichment analysis of candidate TF–TF pairs that are over-represented in enhancer–promoter interactions (61–63), building a predictive model based on TF PPI features is computationally challenging. First, the number of candidate TF PPIs is large (∼200 000). By filtering the features using cell-type specific TF expression, there are still large amounts of potential TF PPI features. Take the human GM12878 cell-line as an example, by only considering TFs that are expressed (17), the number of PPIs between expressed TFs is ∼1900. The excessively high-dimensional TF PPI features easily render predictive models with high overfitting risks. Second, individual TF PPIs are not independent features because of (i) co-binding TF modules along the 1D genome (16)) and (ii) protein complexes consisting of multiple interacting TFs (66,67). Both challenges require advanced feature dimension reduction approaches to efficiently handle the non-linear dependencies in features. In addition, as highlighted by recent benchmark studies (50,51), rigorous settings of cross-validation need to be designed for unbiased performance evaluation and interpretation.

In this study, we developed a new predictive model, ProTECT, to infer long-range enhancer–promoter interactions with substantially improved accuracy. A unique novelty of the model is designing a graph-based dimension reduction algorithm, which can efficiently incorporate combinatorial TF PPI features into the model and, in the meantime, control the overfitting risks. By setting rigorous genomic bin-split cross-validations and controlling various confounding factors, we systematically demonstrated the superior performance of our model compared to existing algorithms. Furthermore, we analyzed the relative importance of TF PPI features in different cell-types and prioritized the key TF PPIs that may participate in the regulation of long-range enhancer–promoter interactions, leading to new mechanistic insights on enhancer regulation. Accordingly, we further classified genes into specific subsets, where enhancer-gene interactions are predicted to be mediated by different TF PPIs. Interestingly, genes in different subsets are enriched with distinct biological pathways, suggesting the specific functional impacts of TF PPIs. Genome-wide implementation of ProTECT in human GM12878 and K562 cell-lines results in 134 792 long-range enhancer–promoter interactions, which are significantly enriched with cis-eQTLs. In addition, by analyzing enhancer–promoter interactions mediated by different TF PPIs, we were able to assign specific TFs as upstream trans-factors to downstream target genes through distal enhancers. Strikingly, the prioritized TF–gene pairs are significantly supported by *trans*-eQTLs, leading to new mechanistic interpretations of trans-genetic effects propagated through the combined regulatory pathways of TF bindings, TF PPIs and long-range chromatin interactions.

## MATERIALS AND METHODS

To predict cell-type specific long-range enhancer–promoter interactions and obtain understandings of the underlying mechanisms, we have developed a new algorithm ProTECT (i.e. PROtein-protein interactions of Transcription factors predicting Enhancer Contacts with Target genes). In addition to cell-type specific multi-omics data, ProTECT (https://github.com/wangjr03/PPI-based_prediction_enh_gene_links) further integrates the information of PPIs between transcription factors as new features, because TF PPIs have been found to be functionally associated with the regulation of chromatin loops (1–5,10,12,13,23). The major steps of ProTECT are summarized in Figure 1E. By creating balanced training sets with confounding factors systematically controlled, ProTECT is trained on cell-type specific chromatin interactions linking distal enhancers and gene promoters. The high-dimensional TF PPI features are hierarchically grouped into feature modules based on a novel graph-based dimension reduction approach. This approach can simultaneously control the overfitting risk and also reveal the cooperative complexes of TF interactions. Our model demonstrated substantially improved accuracy based on a series of rigorous performance evaluations. Along with genome-wide enhancer–promoter interaction predictions, ProTECT also identifies the key TF PPIs involved in chromatin interaction mediation and prioritizes specific gene sets whose expressions are regulated by distinct TF PPIs.

### Chromatin contact maps and multi-omics datasets

ProTECT can take different types of chromatin contact maps as input data (Figure 1E), such as Hi-C (29), Capture Hi-C (30) and ChIA-PET (33). In this study, we used the significant high-resolution Hi-C interactions from human GM12878 and K562 (GEO: GSE63525) (29) to train models for the two cell-lines separately. Enhancer-promoter pairs are labeled as positive samples if overlapping with Hi-C interactions, or are labeled as negative samples otherwise.

Enhancer coordinates are based on Roadmap and ENCODE enhancer annotations (16,17). Cell-type specific enhancer activities in GM12878 and K562 cell-lines are quantified using the cell-type specific DNase-seq signals (17). Other enhancer-associated histone marks, such as H3K27ac or H3K4me1 ChIP-seq data, can also be used to represent enhancer activities and have been found to produce similar predictions in our testing (see Results). Promoters of genes are defined as ±1 kb around transcriptional start sites (TSS), based on gene annotations from GENCODE v17 (68). Cell-type specific gene expressions are measured by RPKM values of RNA-seq dataset from Roadmap Epigenomics project (17). Correlation coefficients are calculated for enhancer-gene pairs across diverse cell-types (16,17) based on the same set of RNA-seq data for genes and DNase-seq data for enhancers.

The ChIP-seq datasets of transcription factor (TF) bindings in GM12878 and K562 are collected from ENCODE separately (16). For each TF, if multiple datasets exist, one ChIP-seq dataset is selected based on data quality evaluations (Supplementary Methods). In total, 129 TFs in GM12878 and 270 TFs in K562 cell-lines are included in the analysis (Supplementary Figure S1A). The significant narrow peaks identified by MACS2 (69) are used to label whether a TF binds to a specific genomic location (Figure 1E). Detailed information of all datasets (i.e. TF ChIP-seq, epigenomic signals, transcriptomic data and chromatin contact maps) are summarized in Supplementary Table S1.

The protein–protein interaction dataset is collected from the STRING database v11 (67). To remove low-quality PPIs, only PPIs with confidence scores greater than 100 in the ‘Experiments’ category are included into the analysis. Multiple PPI confidence score thresholds (e.g. 200 and 300) are also tested, which produce similar predictive performance (see Results). The high-quality PPIs are then summarized into a matrix and represented as a PPI network, where every node corresponds to a protein and every edge corresponds to a protein–protein interaction. To account for the intratypic dimerizations of TFs from the Nuclear Receptor (NR), bHLH and bZIP families, these PPI edges are removed from the PPI network (70) (Supplementary Table S2), because they can only bind locally as dimers. The nodes are further classified into two types: (i) TF protein nodes and (ii) non-TF protein nodes. For edges connecting two TF nodes, i.e. TF–TF PPIs, if both TFs are expressed in the specific cell-type, then the TF–TF PPI is considered as active. Therefore, cell-type specificity is assigned for every TF–TF PPI. non-TF protein nodes are maintained in the PPI network because they are useful to identify indirect TF–TF interactions mediated by non-TF proteins, leading to the discovery of TF PPI modules in subsequent steps.

### Generation of the training dataset and the matrix of features

In a specific cell-type, enhancer–promoter pairs that overlap with significant Hi-C interactions (29), i.e. the enhancer of the pair overlaps with one of the Hi-C interaction anchors and the promoter overlaps with the other anchor, are labeled as positive samples of enhancer–promoter interactions. As reported by previous studies (35,71,72), the data quality of Hi-C interactions whose anchors are located in different topologically associated domains (TADs) are substantially reduced. Therefore, we remove cross-TAD interactions from the analysis, and only use intra-TAD enhancer–promoter interactions, i.e. the interacting enhancer and promoter are located in the same TAD, to train the model.

To avoid biased model training and inflated performance evaluations, we generate a balanced negative set of training samples by randomly selecting the same number of enhancer–promoter pairs that do not overlap with Hi-C interactions. In addition, as pointed out by recent benchmark studies (50), predictions of enhancer–promoter interactions can be substantially biased due to uncontrolled confounding factors. Thus, in the process of generating the balanced random set of negative samples, we strictly control three key confounding factors that have been found to influence the model (Figure 1E): (i) the negative samples of enhancer–promoter pairs should be intra-TAD pairs (Supplementary Figure S1B); (ii) the genomic separation distances between the enhancers and promoters follow the same distance distribution of the positive training set. Uncontrolled genomic distances have been found to substantially dominate the models and result in simple short-range predictions, leading to inflated performance (50,51). Using the positive training set of enhancer–promoter pairs, we group them into different genomic distance bins. For each distance bin (bin-size = 50 kb), we sample the same number of negative enhancer–promoter pairs as observed from the positive set. Therefore, the genomic distance is controlled and the final predictions will not be driven by genomic distances alone (Supplementary Figure S1C, 1D). (iii) The negative enhancer–promoter pairs are sampled for genes which are actively transcribed (Supplementary Figure S1E, F). As demonstrated by previous studies (73), the false negative rates of Hi-C datasets are substantially lower in actively transcribed genomic regions, i.e. more enhancer–promoter interactions can be mapped by Hi-C in active regions compared to repressive genomic regions. To account for this intrinsic bias of Hi-C data, we restrict the sampling of negative enhancer–promoter pairs only from genes whose cell-type specific expression is nonzero (RPKM > 0). By controlling these three key sets of confounding factors, we thus construct the rigorous balanced training dataset for robust model training and performance evaluation. In total, the balanced training dataset contains 5348 enhancer–promoter pairs in GM12878 and 8650 enhancer–promoter pairs in K562.

Based on the cell-type specific multi-omics datasets, the matrix of features are then constructed for enhancer–promoter pairs in the training dataset (Figure 1E). There are three types of features incorporated into the model: (i) activity-based features; (ii) genomic distance and (iii) TF PPI features. Activity-based features include (i) cell-type specific enhancer activity measured by DNase-seq signals as described above (17); (ii) cell-type specific gene expression measured by RNA-seq (17) and (iii) the activity correlations between enhancers and their paired genes calculated from diverse cell-types profiled in the ENCODE and Roadmap Epigenomics projects (16,17). All these activity-based features are differentially distributed across positive and negative training sets, suggesting they are informative to make predictions (Supplementary Figure S2A–C). For each enhancer-gene pair, the genomic distance is calculated as the distance between the center of the enhancer and the gene's TSS. Although they have been controlled in the positive and negative training sets based on genomic bins, there might be residue distance bias within bins. Therefore, the inclusion of genomic distances into the feature matrix captures the residue effects of genomic distances, leading to robust feature prioritization in subsequent analyses.

TF PPIs are the most important set of features for the model because of both the mechanistic relationship with long-range regulation (58,59,74) and their significant enrichment in enhancer–promoter interactions (Figure 1B, C and Supplementary Figure S2D). In each specific cell-type (i.e. GM12878 or K562 cells), all TFs with available ChIP-seq datasets are collected as described above and compared with the PPI database (67). From the pool of all candidate pairs, the TF–TF pairs that are capable of forming direct PPIs are considered as TF PPIs. Considering the differences of binding sites in enhancers or promoters, each TF PPI pair is allocated with two directional features. For example, TF_a_–TF_b_ represents the PPI between enhancer-binding TF_a_ and promoter-binding TF_b_, while TF_b_–TF_a_ represents the PPI between enhancer-binding TF_b_ and promoter-binding TF_a_. Thus, a set of directional TF PPI features is generated. Because the features are generated only for TFs with cell-type specific ChIP-seq signals, PPIs between TFs that are not active in the specific cell-type do not participate in the predictions. Enhancer-promoter pairs are scanned for TF binding peaks in enhancers and promoters. For each enhancer–promoter pair, if TF_a_ binds to the enhancer and TF_b_ binds to the promoter, then the directional PPI feature TF_a_–TF_b_ is labeled as 1. Therefore, a matrix of TF PPI features is constructed for all enhancer–promoter pairs. Combining with the activity-based features and genomic distances, the full matrix of features is then built (Figure 1E).

### Hierarchical TF community detection on the PPI network

Due to the large number of TF PPI features, dimension reduction is fundamentally important for the construction of robust predictive models. Without dimension reduction, there are 1888 TF PPI features in GM12878 and 7066 TF PPI features in K562 cells. Although a number of TF PPIs are enriched in enhancer–promoter interactions (Figure 1B and C), direct incorporation of these TF PPI features makes the model to be over-complicated, leading to poor generalization of predictions. To illustrate the significant overfitting issues of direct incorporation of high-dimensional TF PPI features, a basic random forest model is used to test the performance in GM12878 (29). The features include the activity correlations between enhancers and genes, genomic distances and 1888 active TF PPI features. Although the regular 5-fold cross-validation shows an AUC of 0.89, a rigorous genomic-bin split cross-validation (see subsequent sections on cross-validation) shows the unbiased AUC as 0.55, suggesting strong overfitting problems without advanced feature dimension reductions (Supplementary Figure S3). Thus, a novel predictive model is needed for predicting long-range enhancer–promoter interactions based on PPI features among transcription factors.

To address the over-fitting problem, we substantially reduce the feature dimensions by hierarchically grouping individual TF PPIs into TF PPI modules based on the topology of the PPI network, while maintaining the predictability of the model (Figure 1E). TF PPI modules represent densely connected groups of TFs in the PPI network, and they are hierarchically organized where smaller PPI modules merge together to form larger modules (Supplementary Figure S4). Biologically, using TF PPI modules as features is consistent with the regulatory mechanisms of long-range chromatin loops, because multiple TFs usually interact with each other as protein complexes. Empirically, the biological relevance of TF PPI modules is also supported by the data. As can be seen in Supplementary Figure S5, similar to individual TF–TF pairs, a specific subset of TF modules are strongly enriched in enhancer–promoter Hi-C interactions and are strongly supported by PPI connections (*P*-value = 1.39 × 10^–2^, permutation test).

TF PPI modules are computationally identified from the PPI network (67) using a random-walk based network-community detection approach. The PPI network, including non-TF protein nodes, is modeled as an undirected weighted graph, where the weights on edges are the ‘Experiment’ PPI scores from the STRING database (67). Define $W$ as the adjacency matrix of the PPI network, and define the diagonal degree matrix$\;D\;$as ${D_{ii}} = \mathop \sum \limits_j {W_{ij}}\;$. Hence, based on the stochastic model of random-walks on graphs (75), the 1-step transition probability from node $i$ to node $j$ is $\frac{{{W_{ij}}}}{{{D_{ii}}}}$, and the p-step transition matrix $Tran{s_p}$ can be calculated as $Tran{s_p}\; = \;{({D^{ - 1}}*W)^P}$. Based on the p-step transition matrix, the pairwise distance matrix between TFs (denoted as $R$) can be further calculated as: $R\; = \;diag{(G)^t}*1 + {1^t}*diag( G )\; - 2G$, where $G\; = \;Tran{s_p}*Tran{s_p}^t$. Each entry in the matrix $R$ quantifies the distance between a pair of TFs based on the PPI network structure. Hierarchical clustering is then applied to the pairwise distance matrix $R\;$to identify hierarchical PPI modules of TFs (Figure 1E). ‘wald’ method is used in the hierarchical clustering as suggested by previous studies of network-community detections (76). By testing multiple values (Supplementary Figure S4A and 4B), $p$ is set to be 20 in order to balance the detection of both local (i.e. small-size) and global (i.e. large-size) modules (Supplementary Methods).

In the constructed hierarchical clustering tree, the leaf nodes are individual TF PPIs. By applying the bottom-up merging strategy on the tree, individual TF PPIs are first grouped into small-size PPI modules, *i.e*. S-modules, with the maximum size of ${S_{max}}$. *S*-modules represent densely connected TFs in the PPI network, corresponding to candidate protein complexes. S-modules are further merged to form large-size PPI modules, i.e. *L*-modules, with the maximum size of ${L_{max}}$. *L*-modules represent larger PPI network components that cover multiple densely connected S-modules. Biologically, *L*-modules represent candidate groups of highly interacting protein complexes. The maximum sizes for S-modules (${S_{max}}$) and *L*-modules (${L_{max}}$) are selected based on the modularity score of the clustering (77) (Supplementary Figure S4, Supplementary Methods). The modularity score $Q$ is defined as $Q\; = \frac{1}{{2m}}\;*\mathop \sum \limits_{ij} ( {{W_{ij}} - \frac{{{k_i}{k_j}}}{{2m}}} )*\delta ( {{c_i},{c_j}} )$ where $W$ is the adjacency matrix, ${k_i}$ is the degree of node $i$, $m$ is the total number of edges in the PPI network ($m\; = \frac{1}{2}\;\mathop \sum \limits_i {k_i}$), and ${c_i}$ is the membership assignment to modules for node $i$. Modularity scores are extensively calculated for different choices of maximum module sizes (Supplementary Figure S4C and D), because the choice of specific maximum module sizes automatically determines the total number of modules and results in the final module membership assignments. The optimal size of *S*-modules is selected as the one yielding the maximum modularity score, which guarantees that the generated *S*-modules represent densely connected TF groups. The optimal size of *L*-modules is selected as the one corresponding to the elbow point of modularity score curves, leading to the delineation of large-scale PPI components without significant loss of modularity. Compared to Markov Cluster Algorithm, the PPI modules from our approach demonstrate higher modularity scores and larger module sizes (Supplementary Figure S6), which is desired for feature dimension reductions. Using this procedure, a two-layer hierarchical modular structure is finally built and each individual TF PPI is assigned with the memberships belonging to a specific *S*-module and a specific L-module.

Based on the TF PPI module assignments, individual TF PPI features (i.e. direct TF–TF PPIs) are merged into module-level PPI features, and, therefore, the feature matrix of TF PPIs are restructured accordingly (Figure 1E). There are two types of module-level PPI features: (i) intra-module features, which include all *S*-modules and *L*-modules. The intra-module features cover PPIs between TFs within the same modules. (ii) inter-module features, which include inter *S*-module features and inter *L*-module features. The inter-module features cover PPIs linking TFs from two different modules. Given a pair of *S*-modules, e.g. *S*-module $a$ and S-module $b$, if there exists a TF member from *S*-module $a$ that has PPI with a TF member from *S*-module $b$, then the pair of *S*-modules $a$ and $b$ is included into the feature matrix as one inter *S*-module PPI feature. The inter L-module PPI features are defined in the same way by checking PPIs of TF members from two L-modules. Each inter-module feature is further split into two directional features, depending on the binding sites of TF members in enhancers and promoters. Using this approach, the PPI features are substantially reduced. For example, the 1,888 individual TF PPI features are reduced to only 78 module-level PPI features in GM12878 and the 7066 individual TF PPI features are reduced to only 238 module-level PPI features in K562 cells.

The training set of enhancer–promoter pairs are then scanned for module-level PPI features. For each specific enhancer–promoter pair, based on the counts of individual TF PPI features calculated in the previous step, the counts of module-level PPI features are generated depending on the module memberships of TFs (Figure 1E). For each module-level PPI feature, if multiple TF PPI features are found for an enhancer–promoter pair, the maximum count is used for the module-level feature. Although the number of features is substantially reduced after using module-level PPIs, the specific PPI information is still maintained in this procedure, as shown in Supplementary Figure S5. It suggests that the module-based dimension reduction does not cause the loss of information, while substantially reducing the risk of over-fitting.

### Predictive model of long-range enhancer–promoter interactions

Random forest model is used to predict cell-type specific long-range enhancer–promoter interactions based on the feature matrix constructed above, after module-based dimension reduction (Figure 1E). Random forest model is selected due to its superior performance of handling non-linear feature dependency and its capability of prioritizing the key set of important features for subsequent biological interpretations. As a free model parameter, the number of decision trees in the model is extensively tested with different values, and the accuracy of predictions is found to be robust (Supplementary Figure S7).

Additionally, to quantitatively demonstrate the contributions from TF PPIs, we train random forest models based on two versions of input features: (i) the model is trained using only activity-based features and genomic distances; and (ii) the full set of features including module-level TF PPI features. The Area Under Curve (AUC) values of cross-validations are calculated for the two versions. The increased AUC from version 2 is the quantitative measurement of the additional information contributed from TF PPIs that is not encoded in activity-based or genomic distance features.

### Feature selection

In the random forest model, the backward feature elimination approach is used to select useful module-level TF PPI features, where the features with the minimum importance are recursively eliminated from the model. Furthermore, the statistical significance of the directions of TF PPI features are evaluated. As described in the previous section, every module-level PPI feature is split into a pair of two directional features, based on the binding sites of TFs in enhancers or promoters. For example, the feature *module*$a$*–module*$b$ represents the PPI between an enhancer-binding TF member from *module*$a$ and a promoter-binding TF member from *module*$b$. Reversely, the feature *module*$b\;$*–module*$a$ represents the PPI between an enhancer-binding TF member from *module*$b\;$and a promoter-binding TF member from *module*$a$. Based on the statistical evaluation of the feature directions, insignificant directional features are merged into un-directional features. This feature merging procedure not only reduces the number of features but also reveals the biological roles of TF bindings in the context of different binding orientations.

The determination of whether a pair of directional TF PPI features to be merged into an un-directional feature is a model selection problem. While Akaike Information Criterion (AIC) has been a widely used metric for parametric models, it can not be applied to random forest models, which are non-parametric. Instead, we use the Generalized Degrees of Freedom (GDF) method to calculate a relaxed AIC (78) for the random forest model. GDF is a metric to evaluate the degrees of freedom for Bernoulli distributed data, *e.g*. the binary labels for enhancer–promoter interactions. And it is defined as $GDF \approx \mathop \sum \limits_{\rm{i}} \widehat {({y_{i\;}}^{\prime}} - {\hat y_i})/( {{{y^{\prime}}_i} - {y_i}} )$, where ${y_i}$ is the observed label for data point $i$, $y{^{\prime}_i}$ is the perturbed label by inverting ${y_i}$, i.e. ${y_i}^{\prime}\; = \;1 - {y_i}$, $\widehat {{y_i}}$ is the predicted label from the model using the unperturbed ${y_i}$, and $\widehat {{y_i}^{\prime}}\;$ is the predicted label from the model using the perturbed ${y_i}^{\prime}$. As suggested by previous studies (78), to calculate GDF, 20% samples are simultaneously perturbed. The relaxed AIC of random forest models are then estimated as $AIC\; = \; - 2{l_m} + 2GDF + GDF( {GDF + 1} )/( {N - GDF - 1} )$, where $N$ represents the total number of data points and ${l_m}$ represents the goodness-of-fit of the random forest model. As suggested by previous analyses (78), ${l_m}$ is calculated as the averaged ${R^2}$ value from 5-fold cross-validations.

For each pair of directional TF PPI features, the relaxed AIC metrics are calculated before and after they are merged into an un-directional feature. If a smaller AIC is observed by merging the two directional features, the model with the merged un-directional feature is then selected, because the reduced AIC suggests the directions of the pair are not statistically important. This procedure is conducted for all pairs of directional TF PPI features, and a final random forest model with the selected features is built. In GM12878 cells, the number of module-level TF PPI features is reduced to 53 from 78. In K562 cells, the number is reduced to 139 from 238. This feature selection process further boosts the generalizability of our model and improves the biological interpretations of the learned TF PPI features (i.e. directional or un-directional).

### Cross-validation and performance comparison

To evaluate the performance of our model, i.e. area under curve (AUC), we designed a stringent strategy of 5-fold cross-validation. As highlighted by previous studies (50,51), multiple factors have been found to substantially inflate the performance evaluations and cause overfitting problems. First, the confounding factors (i.e. TAD domain structures, genomic distances between enhancers and promoters, and gene expression levels) need to be controlled. Otherwise, the performance will be biased and dominated by confounding factors. We addressed this issue in the step of data generation as described in previous sections. Negative samples are randomly generated with the confounding factors controlled to have the same distributions as seen from the positive samples. Second, inflated cross-validation AUC can be found due to the spatially proximal enhancer–promoter pairs across the training and testing datasets (50,51). Because TF binding profiles are highly correlated among enhancers and promoters in neighboring genomic regions, proximal enhancer–promoter interactions that are allocated in the testing set will substantially inflate the accuracy. Therefore, random splits of samples based on typical cross-validation may suffer from the dependency of spatially proximal samples allocated in both training and testing sets, as has been noted in previous studies (50,51). To address this issue, we developed a genomic bin-split cross-validation approach (Figure 1E). In this approach, the human genome is first divided into consecutive 1Mb bins. In each of the 5-fold cross-validation steps, 80% of the genomic bins are selected as training bins. And the balanced and confounding factor controlled samples of enhancer–promoter pairs from the training bins are used to train the random forest model. The remaining 20% bins are selected as testing bins, and the samples of enhancer–promoter pairs from the testing bins are used to test the model. Using this genomic bin-split cross-validation method, the dependency between training and testing samples are broken and the model performance can be rigorously quantified.

The performance of our model, ProTECT, is compared with two most recent supervised methods that also leverage TF information: IM-PET (45) and TargetFinder (46). In addition to activity-based features and genomic distances, IM-PET and TargetFinder also includes the TF binding features in enhancers and promoters, while TargetFinder further incorporates TF binding information in the genomic windows between enhancers and promoters. By comparing with these two algorithms, we can further demonstrate the improved accuracy is obtained purely from the unique features of our model, i.e. the PPIs between TFs.

The stand-alone package of IM-PET (https://github.com/tanlabcode/IM-PET) is applied to the same dataset. Since IM-PET automatically makes predictions for all enhancer-gene pairs with distances <2 Mb, only the enhancer-gene pairs overlapping with the dataset are used for performance evaluation, leading to a fair comparison for IM-PET. The TargetFinder software (https://github.com/shwhalen/targetfinder) is also implemented to the same training and testing dataset. The same set of TF ChIP-seq peaks are used to generate the window related features for TargetFinder. 5-fold cross-validation with the same genomic bin-split strategy is applied to remove the potential issues of inflated performance evaluations.

In addition, to quantitatively demonstrate that the improved accuracy of ProTECT is indeed contributed by TF PPI features, we randomly permute the PPIs between TFs, with the degree of each TF in the PPI network unchanged. Furthermore, for every TF, the specific binding sites in enhancers and promoters are also maintained. Therefore, only the TF PPI features are shuffled across enhancer–promoter pairs. The same model training and evaluation procedure are then applied on the permuted dataset. The resulting AUC is then compared to the model trained on the original dataset. This comparison provides direct evidence on the contributions of TF PPIs to chromatin interaction regulation.

### Genome-wide prediction of long-range enhancer–promoter interactions

The trained ProTECT algorithm is applied to all enhancer–promoter pairs with genomic distances <2 Mb across the whole human genome to make genome-wide predictions of cell-type specific enhancer–promoter interactions (Figure 1E). The features for each candidate enhancer–promoter pair are generated in the same way as described in previous sections. By applying the trained random forest classifier, every candidate enhancer–promoter pair is assigned with a predicted score of interacting with each other. To derive unbiased estimates of the statistical significance for the scores, i.e. *P*-values, a null distribution of the scores is generated by permuting the feature matrix across enhancer–promoter pairs. This permutation approach effectively maintains the overall abundances of different features in the shuffled dataset. Based on the null distribution, the *P*-value for each enhancer–promoter pair is then calculated.

Unlike the phase of model training, where the genomic distances are controlled in order to learn specific TF PPI signatures, the phase of genome-wide predictions requires the incorporation of genomic distance information. As shown by chromatin contact maps, e.g. Hi-C datasets, enhancer–promoter pairs with shorter genomic separation distances have higher probability to interact and the probabilities decay as the distances increase (Supplementary Figure S1C). To statistically incorporate the genomic distances based on this prior knowledge, we use the pFDR algorithm (79) to transform *P*-values into distance-aware *q*-values. In pFDR, the distribution of distances between Hi-C linked enhancers and promoters is treated as prior probabilities of interactions for enhancer–promoter pairs. Based on Hi-C data, ProTECT divides the range of distances into consecutive 20 kb bins, and the prior probability of interactions for each distance bin is calculated as:

${\pi _i} = \;5\% *( {number\;of\;significant\;Hi - C\;in\;bi{n_i}} )/$ $( {number\;of\;significant\;Hi - C\;in\;bi{n_1}} )$ , where ${\pi _i}$ is the prior probability for distance-bin $i$. The prior probability for bin 1 (i.e. the shortest distance bin) is set to be the default 0.05. The pFDR under rejection region $[ {0,\gamma } ]$ in distance-bin $i$ is then calculated as $pFDR( \gamma )\; = \;{\pi _i}Pr(P\; \le \;\gamma |H\; = \;0)/Pr( {P\; \le \;\gamma } )\; = \;{\pi _i}\gamma /Pr( {P\; \le \;\gamma } )$, where $P$ represents the *P*-value for each enhancer–promoter interaction. $P$ follows the uniform distribution under the null hypothesis, *i.e*. H = 0, so that $Pr(P\; \le \;\gamma |H\; = \;0)\; = \;\gamma$. $Pr( {P \le \gamma } )$ can be estimated by $\widehat {Pr}( {P \le \gamma } )\; = \;(\mathop \sum \limits_{j\; = \;1}^N \delta ({P_j}\; \le \gamma ))/N$, where ${P_j}$ is the *P*-value for the enhancer–promoter interaction $j$, $N$ represents the total number of *P*-values, and $\delta ( x )$ equals to 1 if x is true and equals to 0 otherwise. Therefore, the *q*-values can be calculated as $Q( P )\; = \;in{f_{\gamma >P}}( {{\pi _i}\gamma /\widehat {Pr}( {P \le \gamma } )} )$, which combines the information from both the distance-aware prior probabilities (${\pi _i}$) and the *P*-values from the random forest model ($P$). Based on the *q*-value threshold of 0.05, the final genome-wide predictions of significant enhancer–promoter interactions are obtained.

### Feature interpretation for mechanistic insights

Using the trained random forest model of ProTECT, we evaluate and rank the importance of features, i.e. the module-level PPI features in the model. The top-ranking module-level PPIs are considered as important features, which represent putative protein complexes that may regulate chromatin interactions. Furthermore, in order to obtain detailed mechanistic understandings of important PPIs between specific TFs, we decode the module-level PPI feature importance into TF-level PPI feature importance. For each prioritized module-level PPI feature, we decompose it into individual TF–TF PPI features, i.e. specific PPIs between an individual enhancer-binding TF and an individual promoter-binding TF. Then the genome-wide predictions of enhancer–promoter interactions are scanned, and the fractions of predictions that contain the specific TF-level PPI features are calculated. The fractions scanned from genome-wide predictions are highly correlated with the fractions calculated from the Hi-C training samples in model training, and are more robust, given the larger pool of genome-wide enhancer–promoter pairs (see Results). Using the fractions, the top-ranking TF-level PPI features are thus identified for each important module-level PPI feature. The prioritized features, both module-level and TF-level, shed light on new biological insights on long-range enhancer regulation.

### Pathway enrichment analysis for genes regulated by specific TF PPIs

To investigate whether chromatin interactions mediated by different TF PPIs may participate in distinct biological pathways, we classify genes based on the specific TF PPI features involved in their interactions with enhancers. For each top-ranking module-level PPI feature, we first identify the top five TF-level PPI features using the method described above. Then, we scan the genome-wide predictions of enhancer–promoter interactions and collect the subset of interactions that contain at least one of the top five TF-level PPI features. Finally, the subset of interactions are ranked by their *q*-values, and the top 1000 genes regulated by these interactions are selected. In this way, the prioritized subset of genes represent strong targets of long-range enhancer regulation mediated by the important TF PPIs. Gene Ontology enrichment analyses are performed on different gene sets using DAVID (80) to check whether they are enriched with specific biological pathways.

### *cis*-eQTL enrichment analysis for predicted long-range enhancer–promoter interactions

As the orthogonal information to validate the accuracy of genome-wide predictions made by ProTECT, *cis*-eQTL datasets from the matched human tissues and cell-types are compared with the predicted enhancer–promoter interactions. Because our genome-wide predictions are made in human GM12878 and K562 cells, we selected four eQTL datasets (81–84) which were profiled from either whole blood tissues or lymphoblastoid cells. A predicted enhancer–promoter interaction is considered to be supported by a *cis*-eQTL (i.e. a significantly associated SNP-gene pair), if the enhancer contains the SNP and the promoter matches with the gene. For each eQTL dataset, the fraction of predicted enhancer–promoter interactions that are supported by *cis*-eQTLs is calculated, and is compared to two versions of negative controls. The first version of negative control is based on random pairing enhancers with promoters that are within 2 Mb distances. The second version of negative control further requires the genomic distances of random enhancer–promoter pairs follow the same distribution from our predicted enhancer–promoter interactions. Therefore, the second version is a more stringent control. For each version, 1000 random samples are generated. And the statistical significance, i.e. *P*-values, of the observed overlapping fractions from our predictions is calculated as the portion of random samples showing a higher overlapping fraction than the real observed one.

In addition to cis-eQTLs, we also use *cis*-hQTLs, i.e. histone QTLs, to evaluate the accuracy of our predictions. The hQTL dataset was also profiled from the human GM12878 cells (85). Similarly, a predicted enhancer–promoter interaction is considered to be supported by a cis-hQTL (i.e. a significantly associated SNP-histone pair), if the enhancer contains the SNP and the promoter overlaps with the histone modification peak. The overlapping fraction is also compared with the two versions of negative controls to justify the enrichment of *cis*-hQTLs in support of our predictions.

### *cis*-eQTL enrichment around TF binding sites

For *cis*-eQTLs that overlap with predicted enhancer–promoter interactions, the genomic locations of the SNPs from *cis*-eQTLs are further compared with TF binding sites within enhancers. Here, the TF binding sites are defined as the ChIP-seq peak summits. For each enhancer included in this analysis, the TFs involved in important PPI features prioritized from the previous steps are selected. The genomic distances between the SNPs and the binding sites of these TFs are calculated. To statistically test whether the SNPs are closer to these important PPI-related TFs, two versions of random controls are generated. The first version is generated by randomly sampling binding sites of any TFs within the same set of enhancers. And the second version is generated by randomly sampling binding sites of TFs that are members of bottom-ranking PPI features, based on feature importance calculations from the previous sections. For each version of negative controls, *P*-values are calculated using Kolmogorov–Smirnov tests by comparing the cumulative distributions of distances.

### *trans*-eQTL enrichment analysis for enhancer-mediated TF–gene pairs

Compared to *cis*-eQTLs, *trans*-eQTLs can provide additional evidence to support the functional associations between the prioritized TFs and specific genes, where the TF’s PPIs are predicted to mediate enhancer–promoter interactions of the target genes. For enhancer-binding TFs that are members of the important PPI features, we first collect the predicted enhancer–promoter interactions mediated by the corresponding PPI features. Genes regulated by these predicted interactions are thus considered as the downstream target genes of the specific enhancer-binding TFs. We define this relationship as enhancer-mediated TF–gene pairs. To exclude the possibility of promoter-mediated effects, we remove the genes whose promoters are also bound by the specific TF.

Using the trans-eQTLs from the published database (86), we identify a subset of trans-eQTLs whose SNPs are located within TF’s gene bodies (plus –10 kb from TSS) and target genes are covered in our input dataset. For this specific subset of *trans*-eQTLs, the SNPs are likely to disrupt the transcription of the TF genes, which in turn affects the TF’s regulation on the downstream target gene's expression (Supplementary Methods).

Hypergeometric test is used to statistically test whether the enhancer-mediated TF–gene pairs significantly overlap with the subset of trans-eQTLs described above. A TF–gene pair is considered to overlap with a *trans*-eQTL if the SNP is located within the TF’s gene body and the gene is the same as the *trans*-eQTL’s target gene. As comparisons, two versions of controls are generated based on the same set of TFs and enhancers. The first version uses the nearest genes to the enhancers as target genes, instead of using ProTECT’s predictions. The second version randomly selects genes within 2 Mb distances as target genes. In each version, the same number of enhancer–promoter interactions are generated as seen from the foreground for each sample, and totally 1000 random samples are created, along with the hypergeometric *P*-values.

## RESULTS

### Long-range enhancer–promoter interaction prediction based on PPIs among TFs

As discovered by recent experimental studies (4–6,8–13,58,59), the protein–protein interactions between specific transcription factors have been found to participate in the regulation of long-range chromatin loops, where the TFs bind to enhancers and promoters respectively (Figure 1A). The PPIs between the enhancer-binding TFs and promoter-binding TFs facilitate the 3D proximity of enhancers and the target gene's promoters. By analyzing the Hi-C interactions between enhancers and promoters in human GM12878 cells, a specific set of TF–TF pairs are found to be enriched in enhancer–promoter interactions (Figure 1B), compared to their frequencies in distance-controlled random enhancer–promoter pairs. Interestingly, these TF–TF pairs are also enriched with known PPIs (Figure 1C, *P*-value = 10^–3^), suggesting that the TFs within each pair can establish interactions at the protein level. Figure 1D shows two examples, where both enhancer–promoter Hi-C interactions contain enhancer-binding CTCF peaks and promoter-binding RUNX3 peaks. And the physical interaction between RUNX3 and CTCF is validated by the PPI database STRING (67), suggesting the RUNX3-CTCF interaction as a putative mechanism linking the enhancers with specific promoters. These observed enrichments strongly indicate the functional importance of TF PPIs in long-range chromatin loops and the possibility of predicting cell-type specific enhancer–promoter interactions using TF PPI features.

Due to the large number of TF PPI features, i.e. PPIs between enhancer-binding TFs and promoter-binding TFs, basic predictive models significantly suffer from overfitting problems, as shown in Supplementary Figure S3. Therefore, to efficiently leverage the information of TF PPIs from the high-dimensional feature space and overcome the overfitting risks, we developed a new machine learning classifier, ProTECT, to predict cell-type specific long-range enhancer–promoter interactions (Figure 1E). Detailed algorithmic designs have been described in Materials and Methods. Overall, there are four main steps to achieve the final predictions: (i) generation of the balanced Hi-C based training dataset, along with cell-type specific TF PPI features; (ii) dimension reduction of features based on hierarchical network community detection; (iii) predictive model construction using random forest and (iv) Genome-wide predictions of cell-type specific enhancer–promoter interactions.

As a new predictive model, here we highlight a series of key novelties of ProTECT (see Materials and Methods for details). First, a rigorous method of controlling confounding factors, such as TAD domains, genomic separation distances and gene expression levels, is designed in the steps of data and feature generations. This method efficiently removes the impacts of confounding factors, which are fundamentally important to control as discussed by recent benchmark analyses (50,51). Second, the graph-based dimension reduction approach not only addresses the potential risk of overfitting but also facilitates the prioritization of functionally important TF PPIs and TF complexes. Third, a generalized degree of freedom (GDF) technique (78) is incorporated to improve feature selections, leading to new biological understandings of specific TFs. Fourth, a stringent genomic bin-split cross-validation strategy is developed for unbiased and robust performance evaluation. This stringent strategy thoroughly breaks the dependency between the training and testing datasets and avoids the inflated performance estimations that have been commonly found in existing methods (50,51). Fifth, a genomic distance-aware pFDR procedure (79) is implemented to identify statistically significant enhancer–promoter interactions along the whole human genome.

We trained ProTECT using the high-resolution Hi-C datasets from the human GM12878 and K562 cell-lines separately (29). The balanced and confounding factor-controlled training dataset contains 5,348 long-range enhancer–promoter interactions in GM12878 and 8650 interactions in K562 cells. The trained classifiers were further applied to make genome-wide cell-type specific predictions of enhancer–promoter interactions. As shown in subsequent sections, the ProTECT algorithm not only improves the prediction accuracy substantially, but also reveals novel mechanistic insights on the functional roles of TF PPIs in the regulation of long-range chromatin loops. The prioritized TFs and their specific PPIs provide a new platform to understand the complex interplay among TFs, enhancers and genes, and remarkably, open a new avenue to systematically interpret both cis- and trans-eQTLs in human genetics analyses.

### Boosted performance based on features of TF PPIs

**Figure 2.**
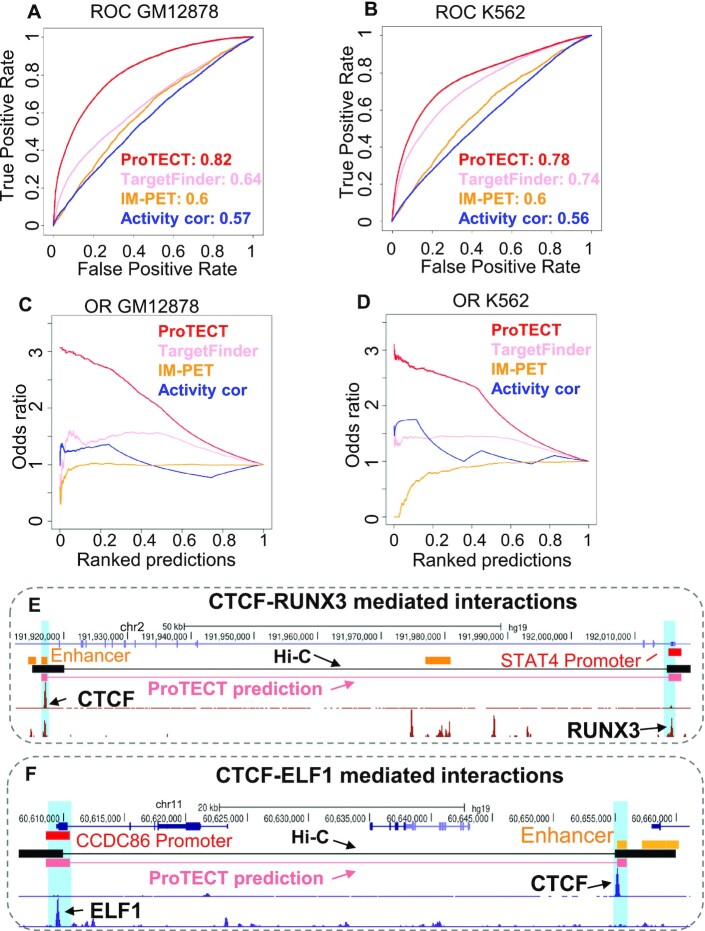
Performance comparison in GM12878 and K562 cells. ProTECT, TargetFinder, and IM-PET are applied on the same input datasets and are evaluated based on the averaged performance of 5-fold genomic-bin split cross-validation. As a baseline comparison, a random forest model using only enhancer-gene activity correlations is also included in the analysis. (**A**, **B**) ROC curves in GM12878 (A) and K562 (B). (**C**, **D**) The enrichment of Hi-C interactions in top-ranking predictions. Cumulative odds ratios of true positives (y-axis), i.e. overlapping Hi-C interactions, are calculated across the ranked lists of predictions where predictions with stronger scores are ranked at the top (x-axis), in GM12878 (C) and K562 (D). (**E**, **F**) Examples of enhancer–promoter interactions predicted by ProTECT (pink paired lines) in GM12878 (E) and K562 (F). In each example, the highlighted enhancer (orange) is predicted to interact with the highlighted promoter (red) by ProTECT. Both predictions are supported by cell-type specific Hi-C interactions (black paired lines). The prioritized TF PPIs mediating the interactions are CTCF-RUNX3 (E) and CTCF-ELF1 (F) respectively, both of which are top-ranking PPI features from the random forest model.

Using the genomic bin-split cross-validation strategy (see Materials and Methods), we rigorously tested the accuracy of ProTECT and compared with the other two supervised methods, i.e. IM-PET(45) and TargetFinder (46). In both GM12878 and K562 cell-lines, ProTECT achieves the highest performance (Figure 2A and B): AUC = 0.82 in GM12878 and AUC = 0.78 in K562 cells. And the accuracy of ProTECT is robust with respect to the number of trees used in the random forest models (Supplementary Figure S7). As comparison, TargetFinder is ranked as the second algorithm with AUC values below 0.74, while the AUC metrics of IM-PET is around 0.6. As a baseline comparison, a random forest model using only activity correlations between enhancers and genes, without using TF PPI features, shows AUC values around 0.57. Because we systematically controlled confounding factors in the training dataset, the AUC estimates are not dominated or biased by those factors, especially the genomic separation distances. Therefore, these comparisons strongly support that the ProTECT model substantially boosts the prediction accuracy over existing algorithms.

In addition to the overall AUC metrics, to demonstrate that ProTECT has better capabilities of pinpointing true enhancer–promoter interactions in top-ranking predictions, we calculated the cumulative odds ratio (OR) of true positives along the ranked list of predictions. As shown in Figure 2C and 2D, ProTECT achieves much higher OR curves than other algorithms, especially in the zone of top-ranking predictions. Because top-ranking predictions are the main *de novo* discoveries used for experimental studies in practice, this observation further exemplifies the superior precision of ProTECT.

Moreover, we further evaluated the robustness of ProTECT’s superior performance with respect to different settings of input features and data. As shown in Supplementary Figure S8, by setting different confidence score cutoffs on PPIs to be included as input features (i.e. 100, 200 and 300), ProTECT robustly achieves the highest accuracy (AUC > 0.78) compared to other methods. In addition, using different epigenetic signals to represent cell-type specific enhancer activity levels, such as DNase-seq, H3K27ac and H3K4me1, ProTECT demonstrates highly similar accuracy, with DNase-seq and H3K27ac based versions slightly better than the H3K4me1 based version (Supplementary Figure S8). Furthermore, we also tested the performance on imbalanced dataset, where the ratio of positive-to-negative samples is 0.1, as suggested by previous studies (45,46). ProTECT consistently shows the best ROC and Precision-Recall curves (Supplementary Figure S9). To obtain orthogonal evidence on ProTECT’s accuracy, we also used a diverse panel of Hi-ChIP (21,87,88) and ChIA-PET (16) datasets from the matched cell-types as gold-standards for enhancer–promoter interactions. Remarkably, ProTECT maintains the highest accuracy across all comparisons based on different gold-standard datasets (Supplementary Figure S10 and 11). Across the five Hi-ChIP evaluations, ProTECT achieves AUC >0.78, while TargetFinder and IM-PET only show AUC <0.66. Using ChIP-PET datasets as gold-standards, ProTECT achieves AUC >0.84 while other methods demonstrate AUC <0.76. These tests systematically support the robustness of ProTECT’s performance advantages.

Figure 2E shows one example predicted by ProTECT in human GM12878 cells. The distal enhancer is located 99.4 kb from the predicted target gene's promoter, and this long-range prediction is supported by a cell-type specific Hi-C interaction (29). Based on the trained random forest model, this enhancer–promoter interaction is mediated by the PPI between the enhancer-binding CTCF and the promoter-binding RUNX3 (Figure 2E). Interestingly, the correlation between the enhancer's activity and the target gene's expression across different cell-types is only 0.28, which strongly suggests the importance of incorporating TF PPI features in predicting enhancer–promoter interactions. A similar example from K562 is shown in Figure 2F, where the distal enhancer is located 46kb from the predicted target gene's promoter, and is also supported by a cell-type specific Hi-C interaction (Figure 2F). This enhancer–promoter interaction, which only shows an activity correlation of 0.261, is successfully predicted based on the PPI between enhancer-binding CTCF and promoter-binding ELF1. Overall, these results demonstrate that TF PPI features can improve the delineation of specific interacting enhancer–promoter pairs from neighboring non-interacting pairs, beyond the information of activity-related features. In addition, specific hypotheses of the mechanisms mediating chromatin interactions, i.e. the functional TF PPIs linking enhancers and promoters, are derived from the model simultaneously.

**Figure 3.**
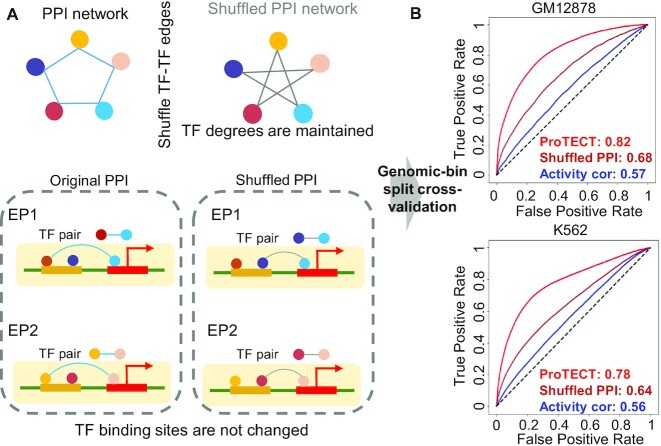
TF PPI features provide additional information beyond TF bindings and activity-based features. (**A**) Schematic figure of the permutation test on TF PPI features. The shuffled PPIs are generated by randomly pairing two interacting TFs from the original pool of TF PPIs, while the degrees of PPI partners and TF binding sites in enhancers and promoters are maintained. Based on the shuffled PPI features, a new random forest model is trained and then evaluated by the same cross-validation procedure. (**B**) ROC plots for the models based on the original TF PPI features (red), the models based on the shuffled TF PPI features (salmon), and the baseline models based on activity-correlation features alone (blue), in GM12878 and K562 cells.

To further justify that the superior performance of ProTECT is indeed due to the information from TF PPI features, we randomly shuffled the TF–TF connections in the PPI network (Figure 3A). Therefore, the specific TF binding sites in enhancers and promoters are strictly maintained (see Materials and Methods), while the PPI features across enhancer–promoter pairs are randomized. This shuffling strategy also controls the degree of PPI partners for each TF, i.e. the number of protein neighbors in the PPI network. By training the ProTECT model on the shuffled data, we found that the accuracy is substantially reduced. The AUC based on PPI-shuffled data is only 0.68, while the original AUC of ProTECT is 0.82 in human GM12878 cells (Figure 3B). Similar decrease of performance is also observed in human K562 cells (Figure 3B). The striking differences of prediction accuracy suggest that the performance improvement of ProTECT is mainly induced by TF PPI features, instead of TF binding information, consistent with previous biological studies of the functional roles of PPIs in chromatin loop regulation (64).

To evaluate the model's dependence on the cell-type specificity of TF bindings, we swapped the TF ChIP-seq data across GM12878 and K562, and run ProTECT based on the swapped data. As expected, the prediction accuracy decreased in both cell-types (Supplementary Figure S12A and B), suggesting the necessity of using TF datasets from the matched cell-types. Interestingly, ProTECT still maintains the highest prediction accuracy when other algorithms are also trained on the swapped TF data, suggesting reasonable generalizability of ProTECT. In addition, to test the model's dependence on the number of TFs included as features, we obtained the intersection subset of TFs whose ChIP-seq are available in both GM12878 and K562, and trained ProTECT based on features derived from this subset. The cell-type specific predictions in GM12878 and K562 demonstrate similar accuracy (AUC = 0.74 and 0.70, Supplementary Figure S12C), suggesting additional TFs are needed in each cell-type beyond the intersection subset.

### Genome-wide prediction of long-range enhancer–promoter interactions

**Figure 4.**
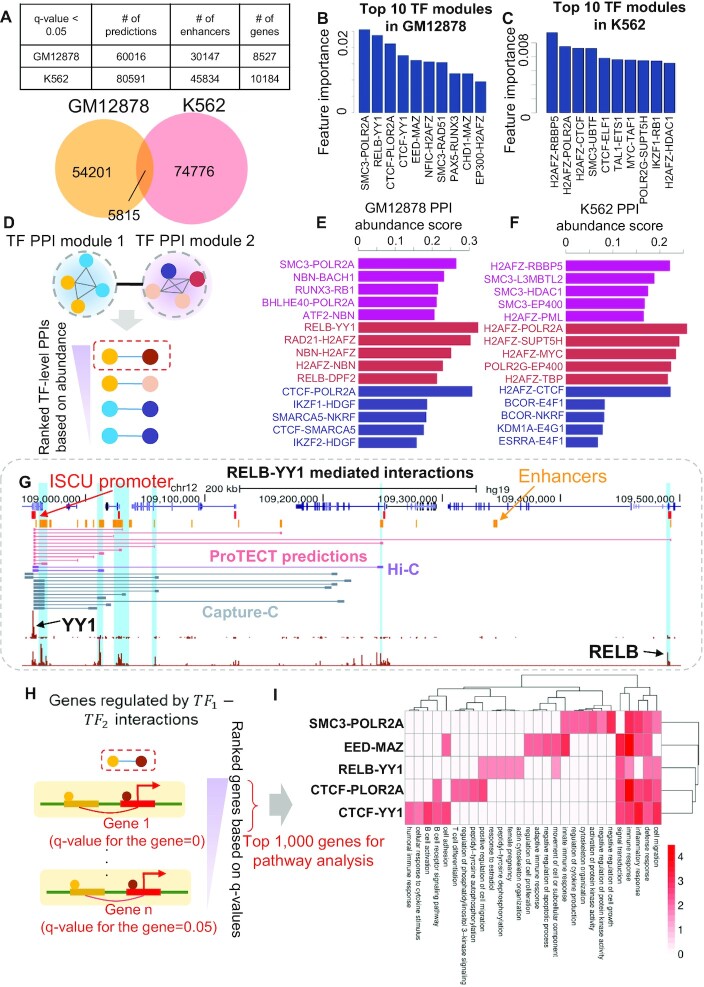
Genome-wide prediction of enhancer–promoter interactions reveals functional roles of TF PPIs in gene regulation. (**A**) Summary of genome-wide predictions in GM12878 and K562. The venn-diagram shows the overlap between predicted enhancer–promoter interactions in GM12878 (yellow) and K562 (salmon). (**B**, **C**) Feature importance (y-axis) of top 10 module-level TF PPI features based on the random forest models in GM12878 (B) and K562 (C). Each module-level PPI feature is named by the most abundant TF-level PPIs between the modules as axis-labels (x-axis). (**D**) Schematic figure of ranking specific TF-level PPIs in each PPI module. For each module-level PPI feature, all TF-level PPIs linking two TFs from the pair of two modules (the pair of modules can be the same to represent intra-module TF-level PPIs) are ranked by their occurrences in the predicted long-range enhancer–promoter interactions (abundance scores). (**E**, **F**) Examples of top 5 TF-level PPIs for three representative module-level features in GM12878 (E) and K562 (F). (**G**) Examples of predicted enhancer–promoter interactions regulated by RELB-YY1 in the ISCU locus. Predicted enhancer–promoter interactions for the ISCU gene are shown as the pink paired lines. Totally 11 enhancers are predicted to interact with the promoter of ISCU, and five predictions are supported by Hi-C (purple paired lines) or capture Hi-C (grey paired lines). ChIP-seq signal tracks of RELB and YY1 (brown signal peaks) are consistent with predictions. (**H**) Schematic figure of ranking enhancer–promoter interactions regulated by specific TF PPIs. For each prioritized TF PPI feature, enhancer–promoter interactions are ranked based on the q-values inferred by ProTECT. Top 1000 genes are then selected by following the ranked list of interactions for pathway enrichment analysis. (**I**) Pathway enrichments of genes regulated by five different TF PPIs in GM12878. The top 10 most enriched pathways for each TF PPI feature are shown. The heatmap is colored based on the –log_10_(*P* value) of pathway enrichments.

The trained random forest model is then applied to the genome-wide dataset in GM12878 and K562 cell-lines separately to predict novel enhancer–promoter interactions (Supplementary Figure S13A–D). All enhancer–promoter pairs within 2Mb distance windows are included into genome-wide predictions (see Materials and Methods), as suggested by observations from experimental Hi-C datasets (29). For each enhancer–promoter pair, a *P*-value from the permutation test is generated, which is further used to derive a *q*-value based on the pFDR approach (79) (see Materials and Methods). Using the *q*-value threshold of 0.05, there are totally 60 016 significant enhancer–promoter interactions predicted in GM12878, and 80 591 significant enhancer–promoter interactions predicted in K562 (Figure 4A). The median separation genomic distance between linked enhancers and promoters is 243 kb in GM12878 (Supplementary Figure S13E), consistent with enhancer's function of long-range regulation. In the predicted GM12878 enhancer–promoter network, >37% of enhancers regulate multiple genes (Supplementary Figure S13F), whose accuracy is consistent with the overall performance (Supplementary Figure S14) and 24% of these multi-gene enhancer links are supported by experimental chromatin interactions. On average, every gene is regulated by 6.9 enhancers (Supplementary Figure S13G), suggesting combinations of multiple enhancers are recruited for precise transcriptional regulation. Similar patterns are also observed in the predicted K562 enhancer–promoter network (Supplementary Figure S13H–J). Furthermore, the predicted enhancer–promoter interactions are highly cell-type specific. By comparing the predictions in GM12878 and K562, only 5815 (∼4.2%) enhancer–promoter interactions are shared by the two cell-types (Figure 4A). Compared to the recent activity-by-contact (ABC) model (89), our genome-wide predictions demonstrate higher accuracy, as quantified by both ROC and Precision-Recall curves, using Hi-ChIP data as gold-standards (Supplementary Figure S15).

### Important protein–protein interactions regulating chromatin interactions

To gain insights of the underlying mechanisms of linking distal enhancers to target gene's promoters, we analyzed the feature importance of module-level PPI features inferred by the random forest model and further prioritize the representative TF-level PPI features. We first identified the top-ranking module-level PPI features, which represent the protein complexes of interacting TFs involved in chromatin loops (Figure 4B and C). For example, in GM12878 cells, module(CTCF)-module(POLR2A) is ranked as the top third feature (here the module-level features are named by the most abundant TF-level PPIs linking the modules). Interestingly, this is consistent with a recent experimental study (90), which also found that the enhancer-binding CTCF interacts with the promoter-binding Pol II and participates in the regulation of long-range chromatin loops. As another interesting example, the module-level PPI feature module(IKZF1)–module(RB1) is one of the top-ranking features in K562, consistent with their critical functions in leukemia cells and their impacts on chromatin structure (91,92). Additional examples of the prioritized module-level TF PPIs are visualized as PPI networks in Supplementary Figure S16, showing the complex PPI connectivity between TF modules binding to enhancers and promoters.

In order to characterize the key PPI features between individual TFs, instead of TF modules, we further decode the module-level PPI features into ranked TF-level PPI features (Figure 4D), based on their occurrences across genome-wide predictions of enhancer–promoter interactions (see Materials and Methods). Genome-wide predictions are used to calculate the abundance scores for TF level PPIs because they provide a large pool of enhancer–promoter links, and the abundance scores are found to be highly correlated with the observations from Hi-C training samples (Supplementary Figure S17, Spearman correlation = 0.95). For each module-level feature, the top 5 most abundant PPI features between specific enhancer-binding and promoter-binding TFs are identified. For example (Figure 4E), RELB-YY1 is predicted to be a key TF-level PPI feature in long-range enhancer regulation. In support of this new discovery, RELB has recently been found to promote gene expression by interacting with YY1 (93). As another example, SMC3-HDAC1 is one of the top-ranking features in K562 (Figure 4F), consistent with the reported regulatory roles of HDAC1 on chromatin structure by interacting with SMC3 (94). The discoveries of these key TFs and their PPIs as candidate functional factors in chromatin loop formation may lead to new biological hypotheses of enhancer regulation for in-depth experimental investigations.

As a demonstration of the potential importance of TF PPIs in linking distal enhancers to promoters, Figure 4G shows the predicted long-range enhancer–promoter interactions for the gene ISCU. There are totally 11 enhancers predicted by ProTECT to interact with ISCU’s promoter, and five of them are supported by experimental data of chromatin interactions based on Hi-C or Capture Hi-C (Figure 4G), indicating the high accuracy of the predictive model. The inferred top-ranking feature is the PPI between enhancer-binding RELB and promoter-binding YY1. Consistent with this prediction, YY1 has a strong ChIP-seq binding site at the promoter of ISCU, and almost all linked enhancers have ChIP-seq signals of RELB binding. Importantly, four out of the five validated enhancers show the strongest RELB ChIP-seq binding signals (Figure 4G), indicating the shared mechanism of these enhancer–promoter interactions for the gene ISCU. In this region, the longest interaction predicted by ProTECT is from a distal enhancer located >547 kb from ISCU’s promoter. Although not captured by chromatin contact map experiments, this specific enhancer contains a sharp ChIP-seq peak of RELB binding (Figure 4G), suggesting this novel prediction as a strong candidate of enhancer–promoter interactions. It also implies the capability of ProTECT to discover long-range enhancer regulation that might be missed by experimental approaches.

To investigate whether the orientations of PPI features between enhancer-binding and promoter-binding TFs have impacts in chromatin interactions, we designed a systematic model selection strategy to test whether a pair of two TF PPI features with opposite directions can be merged into one un-directional PPI feature without reducing the predictive accuracy (see Materials and Methods). Using this approach, 32 pairs of directional PPI features in GM12878 are merged into 16 un-directional features, suggesting there is no statistical preference of binding sites (i.e. enhancers versus promoters) between interacting TFs involved in these PPIs. For example, the features ATF2-SMARCA5 and SMARCA5-ATF2 are merged into an un-directional feature by the model, consistent with the observation that the two directional PPI features have similar abundance in enhancer–promoter interactions (Supplementary Figure S18A). A similar example involves the merge of IKZF1-CREM and CREM-IKZF1 features (Supplementary Figure S18A). In spite of these un-directional PPI features, there are 37 features remaining to be directional in GM12878. For example, there is a significant preference of SMC3-MXI1 feature over the MXI1-SMC3 feature (fold-enrichment = 7.80, Supplementary Figure S18B). This is an interesting observation considering the function of SMC3 (a subunit of cohesin (95)) in chromatin structural maintenance, and the reported regulatory function of MXI1 binding in promoter regions (96). Another example corresponds to the preference of EP300-POL2R2A over POL2R2A-EP300 (fold-enrichment = 9.19, Supplementary Figure S18B), consistent with the well-known enhancer binding activities of EP300 (97) and the transcriptional initiation function of POL2R2A (98). Similarly, 184 pairs of directional PPI features in K562 are merged into 92 un-directional features, while 47 PPI features remain to be directional.

### Genes regulated by different TF PPIs are enriched in distinct pathways

To evaluate the downstream impacts of chromatin interactions mediated by different TF PPIs, we focused on the top 5 module-level PPI features (Figure 4B and C). We identified the strongest enhancer–promoter interactions mediated by each feature separately based on the ranked q-values of predictions (see Materials and Methods). Genes that are regulated by the top-ranking enhancer–promoter interactions are therefore collected for pathway enrichment analysis (Figure 4H). Overall, these prioritized genes are enriched with immune-related or B-cell-related pathways (Supplementary Figure S19A and B), which is expected since the predictions are inferred from GM12878 and K562 cell-lines. Strikingly, for each specific PPI feature, the gene sets are strongly enriched with distinct groups of pathways (Supplementary Figure S19A and B). Figure 4I shows the most enriched pathways for each TF PPI feature discovered in the GM12878 cell-line. Clearly, the enhancer–promoter interactions mediated by different TF PPIs are enriched with diverse biological processes. For example, the CTCF-YY1 feature is found to be associated with long-range regulation of genes in the B cell receptor signaling pathway, while the SMC3-POLR2A feature is associated with genes of the innate immune response pathway (Figure 4I). To exclude the potential bias caused by gene background, we carried out pathway enrichment analysis based on two additional gene backgrounds, respectively: (i) genes with the same set of promoter-binding TFs and (ii) genes with the same set of enhancer-binding TFs (Supplementary Figure S19C and D). Based on these two rigorous gene backgrounds, the majority (>67%) of enriched pathways are still discovered. These differentially enriched pathways further highlight the functional roles of TF PPIs in regulating gene expression and maintaining the specific cellular states.

### Predicted enhancer–promoter interactions are enriched with cis-eQTLs

**Figure 5.**
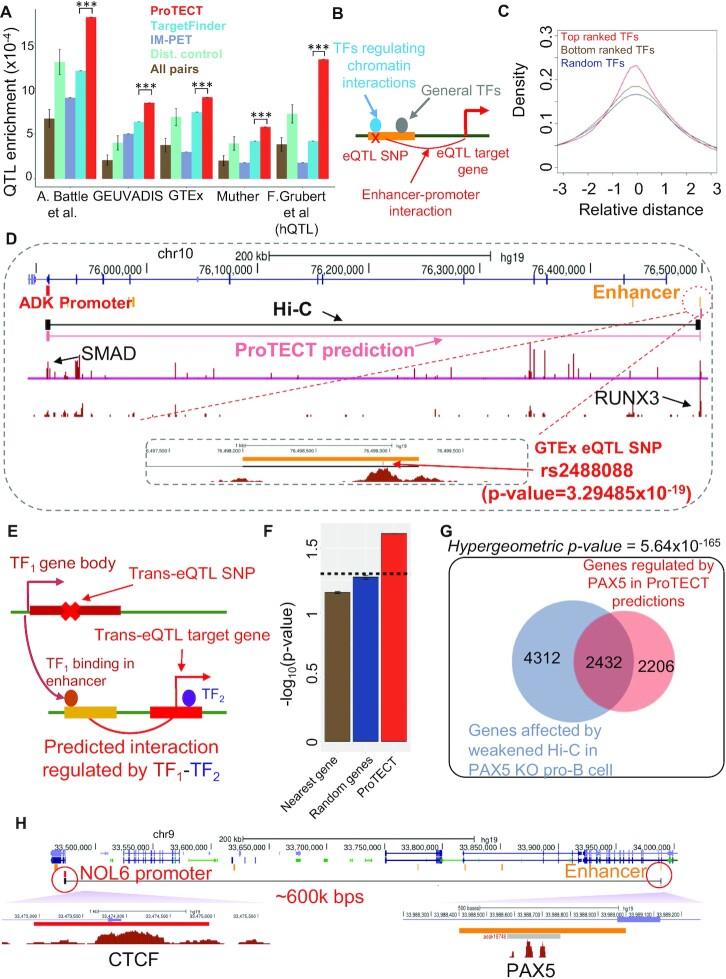
Predicted enhancer–promoter interactions are enriched with cis-QTLs and trans-QTLs. (**A**) *cis*-eQTLs and cis-hQTLs from multiple datasets (x-axis) are significantly enriched in predicted enhancer–promoter interactions in GM12878 (red). The fractions of enhancer–promoter interactions overlapping with *cis*-QTLs (y-axis) are compared with other methods and two versions of controls: (1) random enhancer–promoter pairs (brown) and (2) distance-controlled random enhancer–promoter pairs (blue). 1,000 samples are generated for both versions to calculate *P*-values (***: *P*-value < 1.04 × 10^–4^). Error bars represent sd. (**B**) Schematic figure of *cis*-eQTL SNPs located in the binding sites of functionally important TFs (blue) of chromatin interactions, compared to general enhancer-binding TFs (grey), as a mechanistic hypothesis of cis-regulatory effects on target gene expression. (**C**) Distributions of relative distances between *cis*-eQTL SNPs and binding sites of different enhancer-binding TFs. Relative distances (x-axis) are genomic distances between SNPs and TF ChIP-seq peak summits normalized by the sizes of TF peaks. Binding sites of top-ranking TFs inferred by ProTECT (red) significantly overlap with cis-eQTL SNPs, compared with bottom-ranking TFs (grey, *P*-value = 3.02 × 10^–4^) and random enhancer-binding TFs (blue, *P*-value = 4.17 × 10^–18^). (**D**) Example of a *cis*-eQTL, i.e. the rs2488088-ADK pair, overlapping with a predicted enhancer–promoter interaction (pink paired lines). The predicted interaction is supported by Hi-C (black paired lines). The prioritized PPI feature is RUNX3-SMAD, consistent with the ChIP-seq signal tracks (brown signals). Zoom-in view of the distal enhancer (orange) shows the *cis*-eQTL SNP rs2488088 is located at the peak summit of RUNX3 binding site. (**E**) Schematic figure of *trans*-eQTL SNPs located in specific TF genes, whose binding to enhancers are predicted to mediate long-range enhancer–promoter interactions of *trans*-eQTL target genes. (**F**) Hypergeometric test on the overlaps between *trans*-eQTLs (i.e. trans- SNP-gene pairs) and enhancer-mediated TF–gene pairs, if the SNP is located in the TF’s gene body and the *trans*-eQTL’s target gene is the same as the TF’s target gene (red, *P*-value = 0.014). The –log_10_(*P*-value) (y-axis) from the hypergeometric test is compared to two versions of controls: 1) nearest genes to the enhancers (brown); and 2) random target genes (blue). Each control is generated 1000 times and the error bars show the sd. The black dash line corresponds to –log_10_(0.05). (**G**) Venn diagram comparing genes affected by weakened Hi-C interactions in PAX5 KO pro-B cells and genes regulated by PAX5 in ProTECT predictions (Hypergeometric test, *P*-value = 5.64 × 10^–165^). (**H**) Example of a trans-eQTL, i.e. rs10973104-NOL6 pair, supported by the predicted enhancer-mediated PAX5-NOL6 pair. The predicted enhancer–promoter interaction for NOL6 (black paired lines) is based on the prioritized TF PPI feature PAX5-CTCF. ChIP-seq signals (brown signal tracks) show a strong CTCF peak in the NOL6 promoter (red) and strong PAX5 peaks in the linked enhancer (orange). The *trans*-eQTL SNP rs10973104 is located in the gene body of PAX5, which is 3.6 Mb away from this locus.

Because the predictive model is trained on Hi-C datasets, we use cis-eQTLs as orthogonal evidence to quantitatively evaluate the accuracy of the genome-wide predictions of enhancer–promoter interactions. By comparing the predictions with the SNP-gene pairs of significant eQTLs, we calculated the overlapping enrichment scores (see Materials and Methods). Using four eQTL datasets generated from matched cell-types or tissues (e.g. whole blood tissues or lymphoblastoid cell-lines) (81–84), the predicted enhancer–promoter interactions in GM12878 cell-line show significantly higher fractions overlapping with eQTLs, compared to stringent distance-controlled random interactions and other algorithms (*P*-value < 1.04 × 10^–4^, Figure 5A). Similar, but relatively weaker, enrichment with eQTLs is found for predictions in K562 cell-line (Supplementary Figure S20A). In addition to *cis*-eQTLs, we compared our predictions in GM12878 with histone-QTLs from the same cell-line (85) and also observed strong enrichment (*P*-value = 3.27 × 10^–5^) compared to distance-controlled random samples and other algorithms (Figure 5A). These observations not only support the high accuracy of genome-wide predictions but also suggest the putative mechanisms of *cis*-eQTLs mediated by chromatin interactions between regulatory elements and target genes.

### *cis*-eQTLs are enriched in binding sites of prioritized TFs

The prioritized TF PPI features by the ProTECT model provides a new metric of delineating functionally important TFs for enhancer regulation against general enhancer-binding TFs, which is complicated due to the large array of TFs binding to enhancers. For a typical enhancer, it contains 10 different TF binding sites on average, based on the counts of TF ChIP-seq peaks in GM12878 from the ENCODE project (16). However, binding itself is not sufficient to assign functional importance for TFs. As found by previous studies, TFs binding in enhancer regions are not equally important for the function of enhancers, with many enhancer-binding TFs lacking evidence of regulatory impacts on gene expression (99). This ambiguity hinders the understanding of enhancer activation and downstream effects. We hypothesized the TFs involved with top prioritized PPI features are more likely to be functional for enhancers. We tested this hypothesis by checking the enrichment of *cis*-eQTL SNPs within the binding sites of the prioritized TFs in enhancers (Figure 5B, see Materials and Methods). The *cis*-eQTLs are called in whole blood tissues from the GTEx project (81). Interestingly, the SNPs of *cis*-eQTLs are located significantly closer to the binding sites of prioritized TFs in GM12878 (*P*-value = 4.17 × 10^–18^, Kolmogorov–Smirnov test), compared to the binding sites of other adjacent enhancer-binding TFs (Figure 5C). To control the potential bias caused by data availability, we also generated a more stringent background only using TFs included in the model but inferred with low feature importance (see Materials and Methods). Compared with this new background, the prioritized TFs are still significantly enriched with cis-eQTL SNPs (*P*-value = 3.02 × 10^–4^, Kolmogorov-Smirnov test, Figure 5C). In the K562 cell-line, cis-eQTL SNPs are also closer to the binding sites of the prioritized TFs but not statistically significant (Supplementary Figure S20B). Overall, this analysis supports the stronger regulatory effects of prioritized TFs whose PPIs may mediate long-range enhancer–promoter interactions. Additionally, the prioritized TF binding sites provide a new layer of information to pinpoint regulatory SNPs at a higher resolution, by dissecting the ambiguity of numerous TF bindings within enhancers.

As a representative example, a distal enhancer located > 589kb away is predicted by ProTECT to interact with the promoter of the ADK gene in GM12878 (Figure 5D), which is supported by experimental Hi-C data (29). This long-range interaction is also supported by a significant eQTL, i.e. rs2488088-ADK (*P*-value = 3.29 × 10^–19^) (81). The prioritized TF PPI feature for this interaction is RUNX3-SMAD, where RUNX3 binds to the enhancer and SMAD binds to the promoter. By zooming into the enhancer element, which is 1.2 kb long and contains binding sites of five different TFs, the SNP rs2488088 is found to be precisely located at the ChIP-seq peak summit of RUNX3 (Figure 5D), consistent with our prioritization of RUNX3 as the important TF for this enhancer. This observation also implies the mechanistic interpretation of this non-coding SNP, whose disruptive effect on the RUNX3 binding causes the loss of RUNX3-SMAD mediated long-range interaction to ADK.

### *trans*-eQTLs are enriched in enhancer-mediated TF–gene pairs

As one of the advantages of the ProTECT algorithm, both *cis*-regulatory elements (i.e. enhancers) and *trans*-regulatory factors (i.e. TFs) are jointly modeled in long-range chromatin interactions. In traditional studies of *trans*-regulation of gene expression, analyses have been mainly limited to promoter-binding TFs as candidate *trans*-regulatory factors (100,101). Based on the functional impacts of the predicted important TF PPI features (Figure 4B-I) and the observed enrichment of *cis*-eQTL SNPs in prioritized enhancer-binding TFs (Figure 5B–D), we hypothesized that there is an enhancer-mediated pathway of *trans*-regulation, i.e. the enhancer-binding TFs associated with top-ranking PPI features for long-range chromatin interactions are *trans*-regulatory factors for the expression of distal target genes (Figure 5E). To quantitatively validate this hypothesis, we compared the enhancer-mediated TF–gene pairs with significant *trans*-eQTLs (86), and the significance of overlaps are statistically tested using Hypergeometric tests (see Materials and Methods). Interestingly, the enhancer-mediated TF–gene pairs are found to be strongly supported by *trans*-eQTLs (*P*-value = 0.014, Figure 5F, Supplementary Figure S20C), suggesting that the SNPs of *trans*-eQTLs are associated with target gene's expression via the disruption of the TF gene's activity (Figure 5E), although the SNPs may be located far away from the target genes or even located in different chromosomes. The observed statistical significance is also stronger than two versions of controls, excluding the potential confounding effects of biased enhancer activity and genomic distances (Figure 5F, see Materials and Methods).

To obtain additional experimental evidence on the predicted enhancer-mediated TF–gene regulation, we leveraged a differential Hi-C interaction dataset in mouse pro-B cells where 7810 weakened Hi-C interactions were identified following PAX5 knock-out (102). The top-ranking PAX5 related PPI feature predicted by ProTECT is PAX5-CTCF, consistent with their collaborative roles in B cells (103,104). Based on our genome-wide predictions in GM12878, we identified the subset of PAX5-CTCF mediated enhancer–promoter interactions (see Materials and Methods), and thus collected the enhancer-mediated target genes of PAX5. To purify the subsequent analysis, genes whose promoters are also bound by PAX5 are removed from the list. If PAX5 is a true trans-regulatory factor for these genes, the genes are expected to be targeted by the weakened long-range interactions following PAX5 knock-out. By mapping the genes to their homology in the mouse genome (105), 6,744 enhancer-mediated target genes of PAX5 are conserved. Strikingly, these genes are found to significantly overlap with the genes of weakened Hi-C interactions in PAX5-/- pro-B cells (102) (hypergeometric *P*-value = 5.64 × 10^–165^, Figure 5G). To control the potentially biased enhancer activity and TF bindings, we generated two versions of controls. The first version randomly selects genes as enhancer-mediated target genes of PAX5. And the second version randomly chooses target genes of other TFs. 1000 random samples are generated for each version and the same number of genes are selected for each sample. Both versions of negative controls show decreased overlap with genes of weakened Hi-C interactions in PAX5–/– pro-B cells (*P*-value = 10^–3^), supporting the predicted *trans*-regulatory links between PAX5 and target genes by ProTECT. Figure 5H shows one representative example of PAX5-CTCF mediated long-range enhancer–promoter interaction (∼600 kb), where the enhancer contains multiple PAX5 binding sites and the promoter of the target gene, *i.e*. NOL6, contains a strong CTCF binding site. Interestingly, NOL6 is linked with weakened Hi-C interactions in PAX5–/– pro-B cells. These strong experimental validations, along with the enrichment of *trans*-eQTLs, suggest the biological validity of the predicted enhancer-mediated TF–gene pairs, and provide a new regulatory mechanism to discover and interpret *trans*-regulatory genetic variants.

## DISCUSSION

In this study, we have developed a novel supervised algorithm, ProTECT (https://github.com/wangjr03/PPI-based_prediction_enh_gene_links), to predict long-range enhancer–promoter interactions. By incorporating new features of protein–protein interactions among transcription factors, the algorithm achieves superior performance compared to other methods, based on a rigorously designed genomic bin-split cross-validation procedure. Considering the overfitting risk of high-dimensional inter-dependent TF PPI features, a novel network-community based dimension reduction strategy is used to hierarchically organize TF PPIs into module-level features. This approach efficiently improves the generalizability of the predictive model to make robust predictions based on complex TF PPI patterns, while maintaining the detailed ranking of TF-level PPI features for specific mechanistic understandings of long-range enhancer regulation. With the impacts of confounding factors strictly controlled, the relative contributions of different features are systematically evaluated, which shows that TF PPIs contain substantially additional information beyond activity-based features of enhancers and genes.

The genome-wide implementation of ProTECT in GM12878 and K562 cell-lines generated 60 016 and 80 591 new predictions of significant enhancer–promoter interactions, which will be useful resources of cell-type specific enhancer regulation for biologists. In addition, a set of prioritized TF PPIs, in both module-level and TF-level, are identified as the key PPIs mediating long-range chromatin loops. Different TF PPIs are found to mediate enhancer regulation for genes in distinct biological pathways, implying specific functional roles of complex TF cooperation. The TF members participating in these prioritized PPI features can be used as candidate targets for knock-out to investigate the changes of specific enhancer–promoter interactions, which will expand the insights on the underlying mechanisms of chromatin loop formation and long-range gene regulation.

To gain orthogonal evidence of the validity of genome-wide predictions, *cis-* and *trans*-eQTLs are compared with the predicted enhancer–promoter interactions in three ways, each of which supports one aspect of the interplay among TFs, enhancers and genes. First, the enrichment of overlaps between *cis*-eQTLs and enhancer–promoter interactions suggests the accuracy of predicted long-range *cis*-regulation by distal enhancers. Second, the enrichment of *cis*-eQTL SNPs located within the binding sites of prioritized TFs underscores the precise delineation of functionally important TFs for enhancer activities against other general enhancer-binding TFs. Third, the enrichment of overlaps between *trans*-eQTLs and enhancer-mediated TF–gene pairs highlights the novel identification of *trans*-regulatory pathways from upstream TFs to downstream genes via distal enhancers. The promising enrichment analyses further indicate that the predictions from ProTECT can be used as a platform to interpret *cis*- and *trans*-eQTLs, i.e. characterize the non-coding SNP’s disruptive effects propagated through long-range enhancer regulation on gene expression. Therefore, combined with eQTL datasets, the ProTECT model can also be a useful tool to generate testable hypotheses in statistical genetics studies.

To control the model complexity, only direct PPIs between TFs are included as features, while indirect PPIs between TFs may also participate in the regulation of chromatin loops. For example, an enhancer-binding TF and a promoter-binding TF may not be able to interact with each other but they both can interact with a third protein. The incorporation of module-level TF PPI features helps to capture the potential indirect PPIs to some degree, but does not explicitly address this problem. Due to the large number of indirect PPI features and the limited number of labeled samples for model training, more advanced designs of feature selection will be needed to achieve a balance between predictive accuracy and model generalizability.

As a major novelty of the ProTECT model, the efficient inclusion of TF PPIs as features not only improves the predictions but also reveals mechanistic insights on long-range enhancer regulation. In the meantime, the algorithm requires the availability of large panels of TF ChIP-seq data for the specific cell-types under study, which may be a practical challenge for users. As one of the directions to extend the ProTECT model, it is possible to leverage the combined information of chromatin accessibility data, e.g. DNase-seq or ATAC-seq data, and TF binding motif annotation datasets as approximations for cell-type specific TF bindings. Several recent studies have demonstrated the reasonable accuracy of this approximation (16,17). Furthermore, multiple imputation algorithms have been recently developed for ENCODE cell-types or tissues to impute cell-type specific TF binding ChIP-seq signals (106,107). The imputed TF binding signals can be used as alternative inputs for the model to make cell-type specific predictions of enhancer–promoter interactions, for cell-types lacking ChIP-seq datasets. As an evaluation of this possibility, we generated the imputed TF bindings by overlapping TF motifs with cell-type specific DNase-seq peaks, and then derived TF PPI features based on the imputed data. Remarkably, applied on the imputation-based input features, ProTECT is able to achieve high accuracy (Supplementary Figure S21). This evaluation strongly supports the wide applicability of ProTECT on diverse cell-types even if TF ChIP-seq data is not directly available.

## DATA AVAILABILITY

ProTECT is an open source infrastructure available in the GitHub repository (https://github.com/wangjr03/PPI-based_prediction_enh_gene_links). The genome-wide predictions of enhancer–promoter interactions in human GM12878 and K562 cell-lines, including both hg19 and hg38 versions, are also available in this GitHub repository. Testing datasets, including input feature matrices and expected predictions, are also provided.

## Supplementary Material

gkab841_Supplemental_Files
