# Supplementary material for: Predict long-range enhancer regulation based on protein–protein interactions between transcription factors
Source: Nucleic Acids Res. 2021 Sep 27;49(18):10347–68. doi: 10.1093/nar/gkab841 (PMC8501976; doi:10.1093/nar/gkab841)
Supplement: gkab841_Supplemental_Files [file gkab841_supplemental_files.zip › Supplementary_Figures_1-21_Supplementary_Methods.pdf]

A

Summary of input data			
	TF	Epigenetic mark	RNA-seq
GM12878	129	1	1
K562	270	1	1

Summary of TF features		
	PPIs	Module interactions
GM12878	1888	53
K562	7066	139

Balanced training sets with confounding factors controlled:

- ✔ **Intra-TAD;**
- ✔ **Same distance;**
- ✔ **Active genes;**

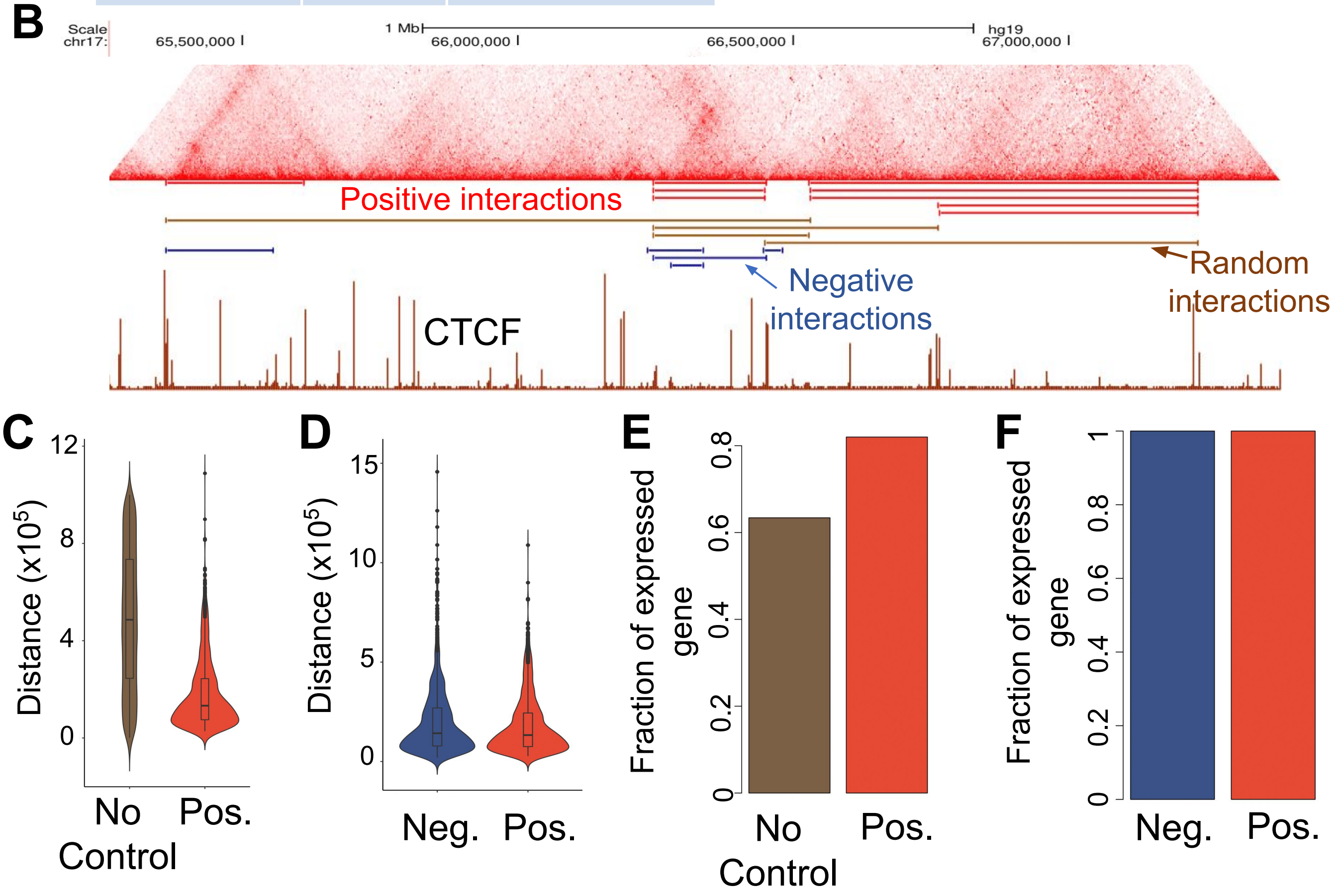

Supplementary Figure 1: Summary of training dataset generation and confounding factor controls. (**A**) Summary of the multi-omics datasets (upper panel) and the numbers of TF PPI features (lower panel). The number of TF features are reduced by applying the hierarchical network community detection on the PPI network. (**B-F**) A balanced training dataset is generated by controlling three sets of confounding factors. (**B**) Inter-TAD enhancer-promoter interactions are removed. Compared with Hi-C interactions (red lines), randomly generated enhancer-promoter interactions (brown lines) are enriched with inter-TAD pairs and cover domain boundaries. Consequently, the stronger binding strength of boundary-enriched TFs, e.g. CTCF, between enhancers and genes are observed for negative interactions. The inter-TAD pairs are removed after confounding factor control (blue lines). (**C-D**) The genomic distance is controlled. Compared with the genomic distance of positive interactions (red), (**C**) Genomic distances of randomly paired negative enhancer-promoter interactions (brown) are longer than positive interactions observed from Hi-C (red), before confounding factor controls. (**D**) The genomic distance distribution of negative sets (blue) is consistent with positive interactions (red), after confounding factor control. (**E**) The fraction of linking to expressed genes in random enhancer-gene interactions (brown) is lower than the fraction in the positive set based on Hi-C (red), before confounding factor control. (**F**) All target genes are expressed in both negative interactions (blue) and positive interactions (red), after confounding factor control.

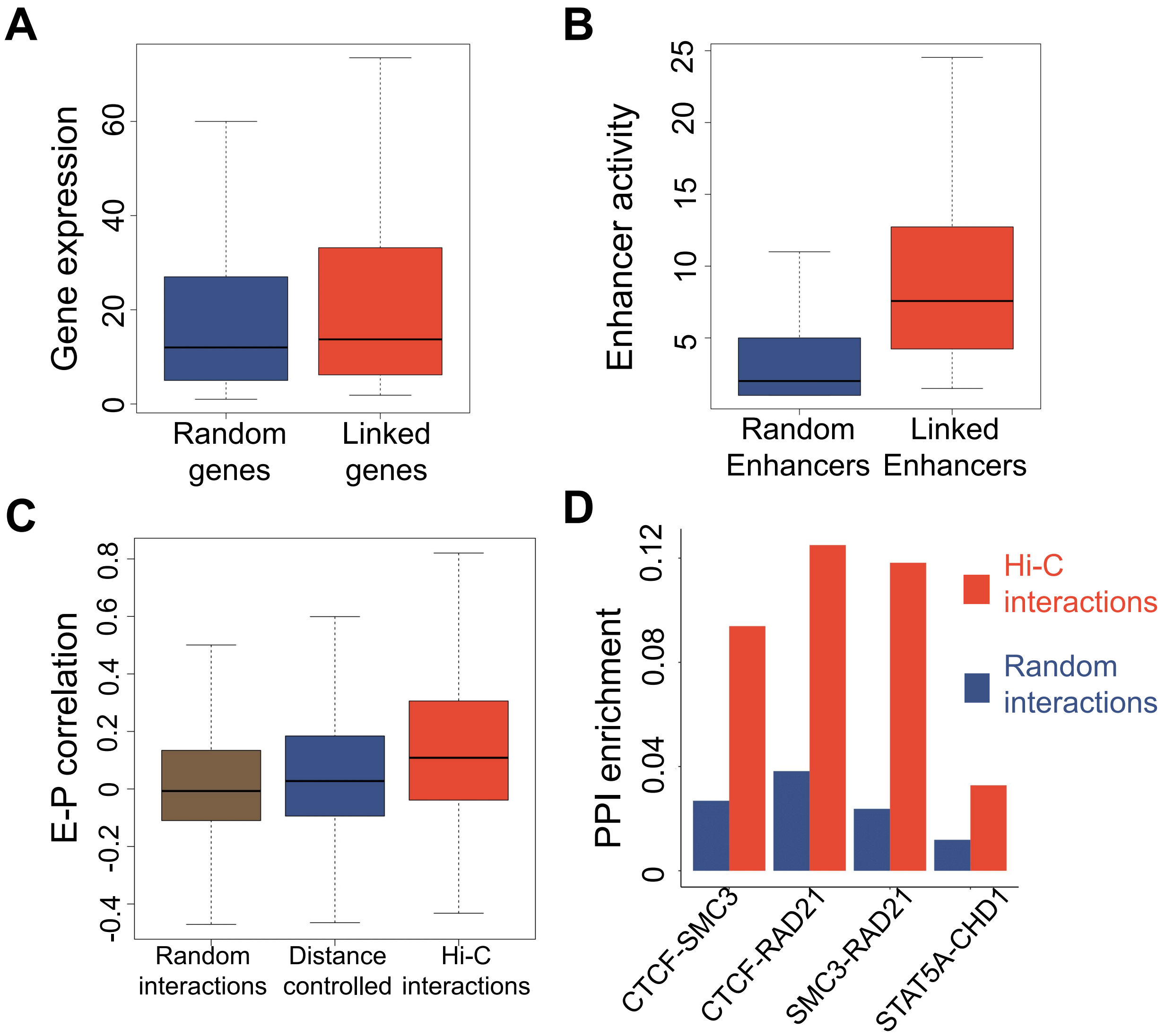

Supplementary Figure 2: Predictive powers of features are supported by the differential distributions of features in Hi-C based positive interactions and random negative interactions. (**A**) The genes linked by Hi-C interactions (red) have a higher expression level than random genes (blue). (**B**) The enhancers linked by Hi-C interactions (red) are more active than random enhancers (blue). (**C**) The enhancer-promoter interactions overlapping with Hi-C interactions (red) have higher activity correlations between linked enhancers and genes across 56 cell-types. As comparisons, two versions of controls are generated: 1) Distance controlled random enhancer-promoter pairs (blue): randomly paired enhancer-promoter interactions which follow the same distance distribution as Hi-C interactions. 2) Random pairs (brown): randomly paired enhancer-promoter interactions without controlling any confounding factors. For each version, the same number of enhancer-promoter interactions are generated and the correlations between enhancer activity and gene expression across cell types are calculated. (**D**) Examples of TF PPIs showing differential enrichments in Hi-C interactions (red) vs. random interactions (blue). The PPI enrichment is calculated as the fraction of enhancer-promoter interactions containing the specific TF PPI features.

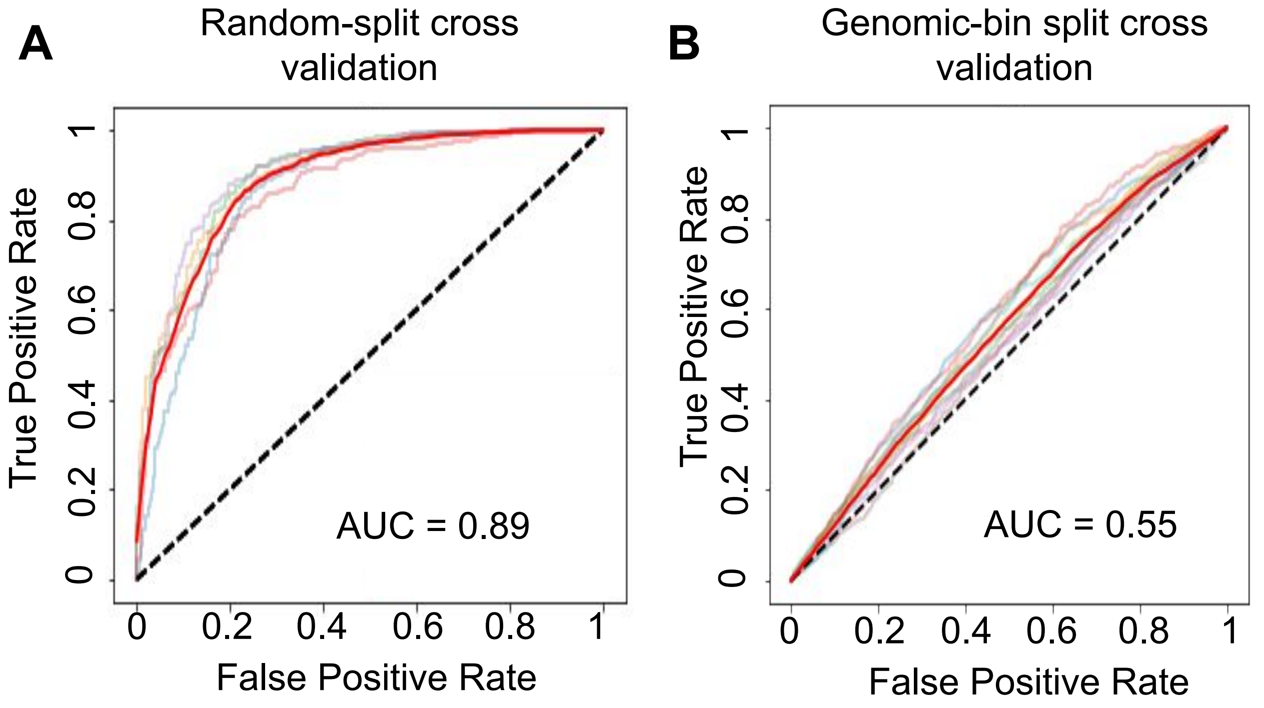

Supplementary Figure 3: Advanced feature dimension reduction is needed due to the risk of overfitting. (**A**) The ROC curves of a random forest predictive model using high-dimensional TF PPI features, based on typical cross-validation. (**B**) The ROC curves of the same predictive model, based on the rigorous genomic-bin split cross-validation, where the dependency between the training and testing datasets are strictly broken. The significantly decreased AUC is due to the large number of TF PPI features, suggesting advanced feature dimension reduction approach is required to construct a robust predictive model.

A

p	Module count (GM12878)	Module count (K562)
10	831	1083
20	401	526
30	134	419

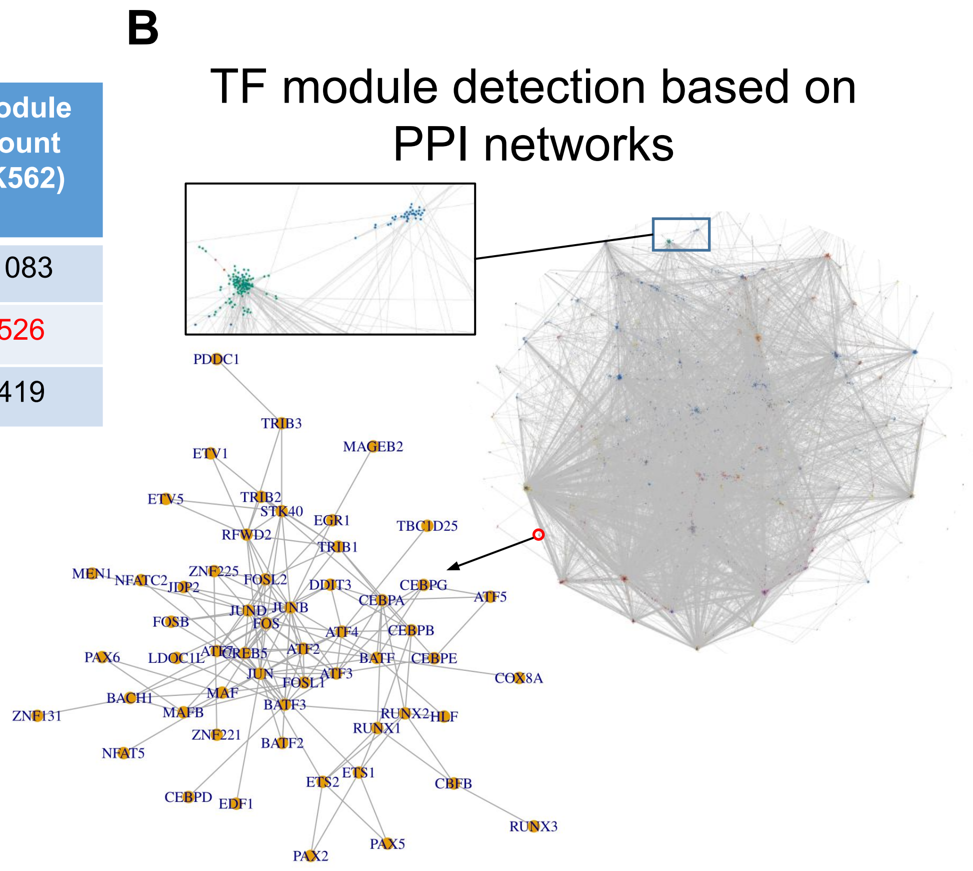

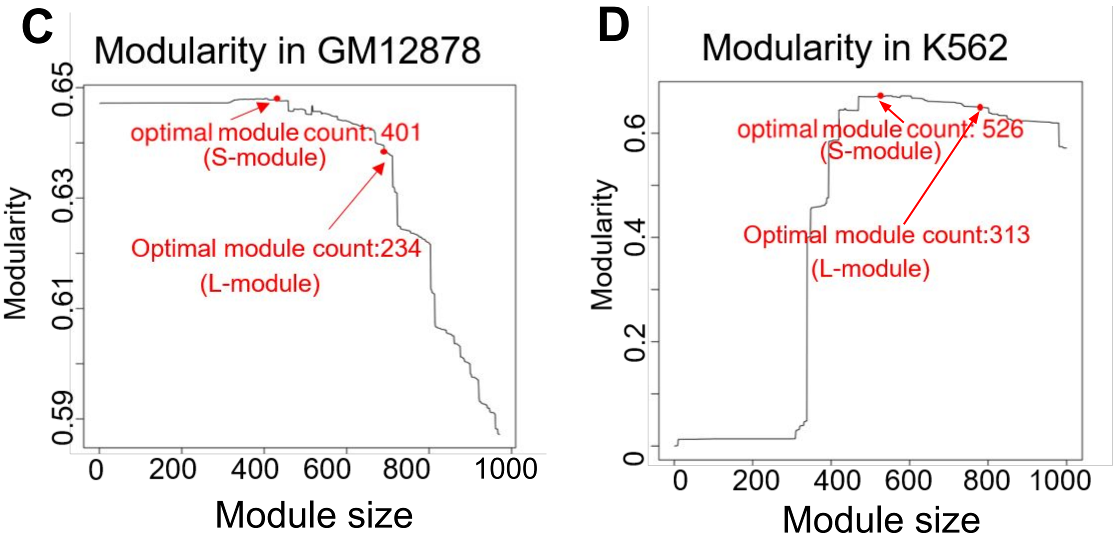

Supplementary Figure 4: Hierarchical network-community detection based on the PPI network to construct module-level TF PPI features. (**A**) The number of TF PPI modules as a function of different random-walk step-sizes (P). step-size=20 is selected to balance the detection of local and global modules. (**B**) Examples of detected TF PPI modules. Nodes represent proteins and edges represent PPIs. TFs belonging to the same module are annotated with the same color. (**C-D**) Modularity scores of hierarchical network-community detections with different maximum module sizes. The optimal number of S-modules is selected based on the highest modularity score. The optimal number of the L-module is selected based on the elbow points of the modularity score curves.

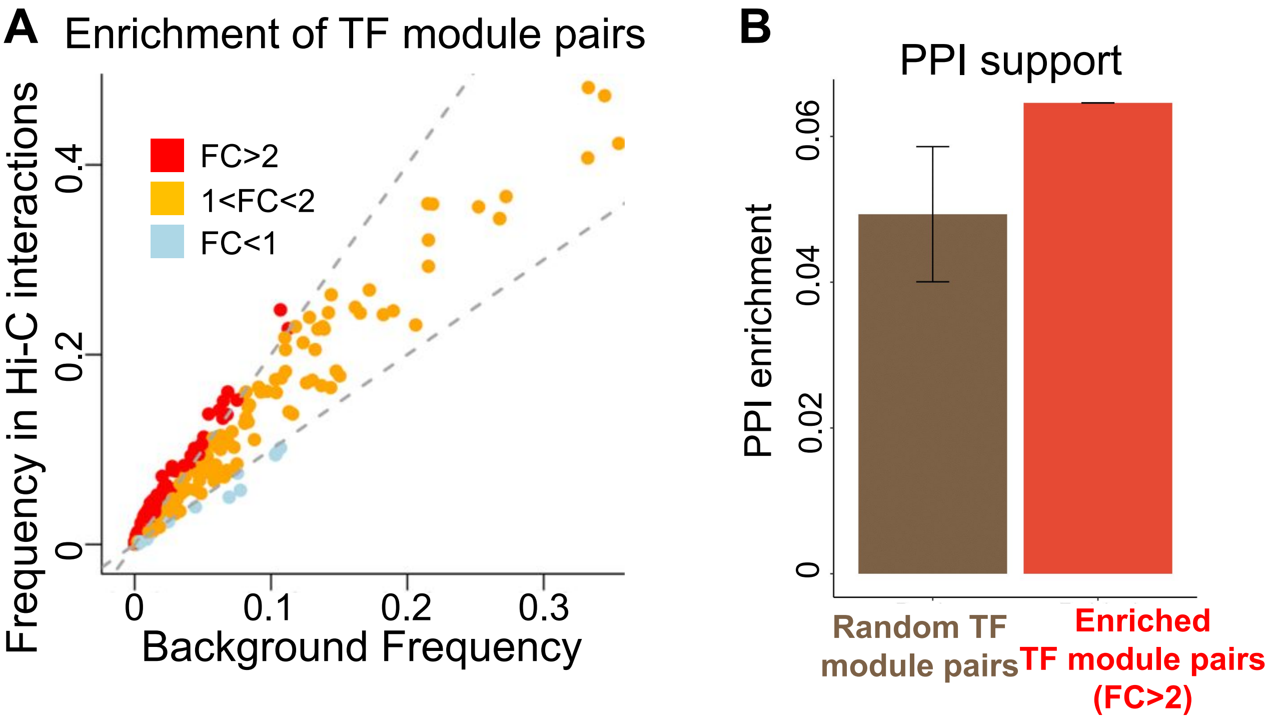

Supplementary Figure 5: Enrichment analysis and PPI support analysis for TF module pairs. (**A**) The enrichment of TF module pairs in Hi-C interactions (y-axis) compared to background random interactions (x-axis). Points represent TF module pairs. Frequency is calculated as the fraction of enhancer-gene interactions containing the specific TF module pairs, one on the enhancer side and one on the linked promoter side. Fold-change (FC) is the ratio of the frequency in Hi-C interactions over the frequency in backgrounds. TF module pairs are colored by the FC (red: FC>2; orange: 1<FC<2; blue: FC<1). (**B**) Enriched TF module pairs are supported by inter-module PPIs. The fraction of pairs supported by inter-module PPIs is calculated for the set of enriched TF module pairs (red). As controls, the TF members from the enriched module pairs are randomly paired (brown). An empirical statistical test is done based on 1,000 random repeats of controls (p-value=1.39×10^{-2}).

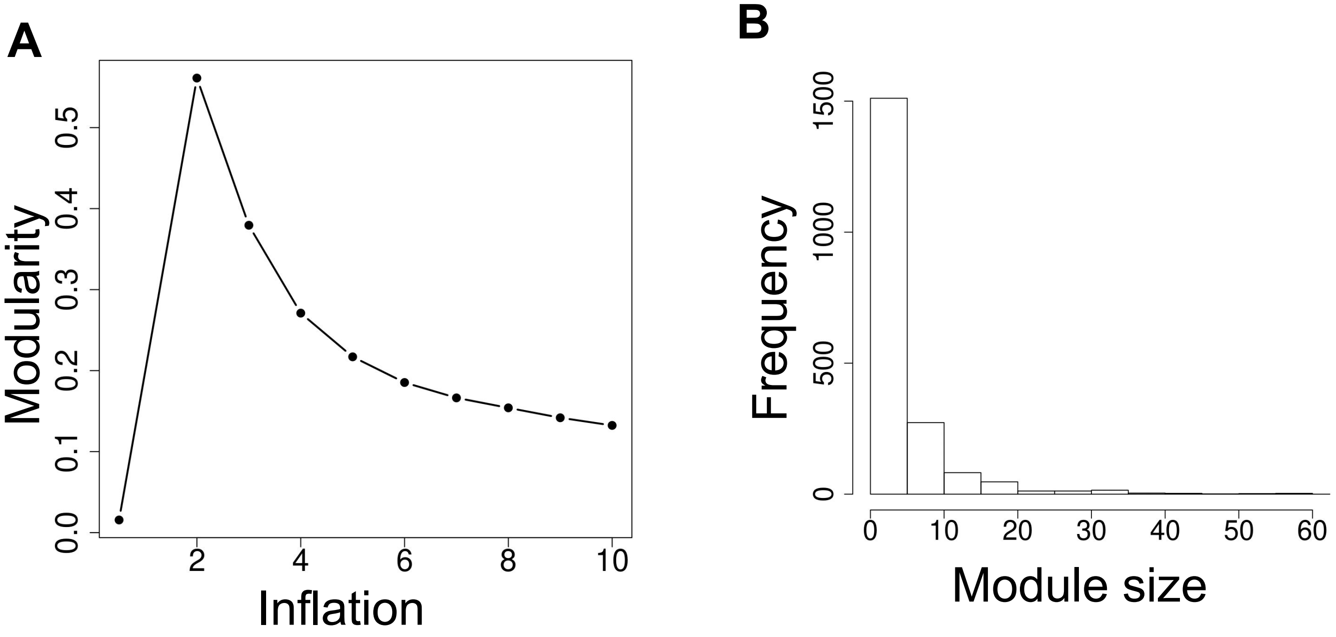

Supplementary Figure 6: PPI community detection based on the Markov Cluster Algorithm (MCL). (**A**) Modularity scores of PPI communities using MCL with different inflation values. (**B**) Distribution of the module sizes based on the MCL prediction using the inflation value with the highest modularity score.

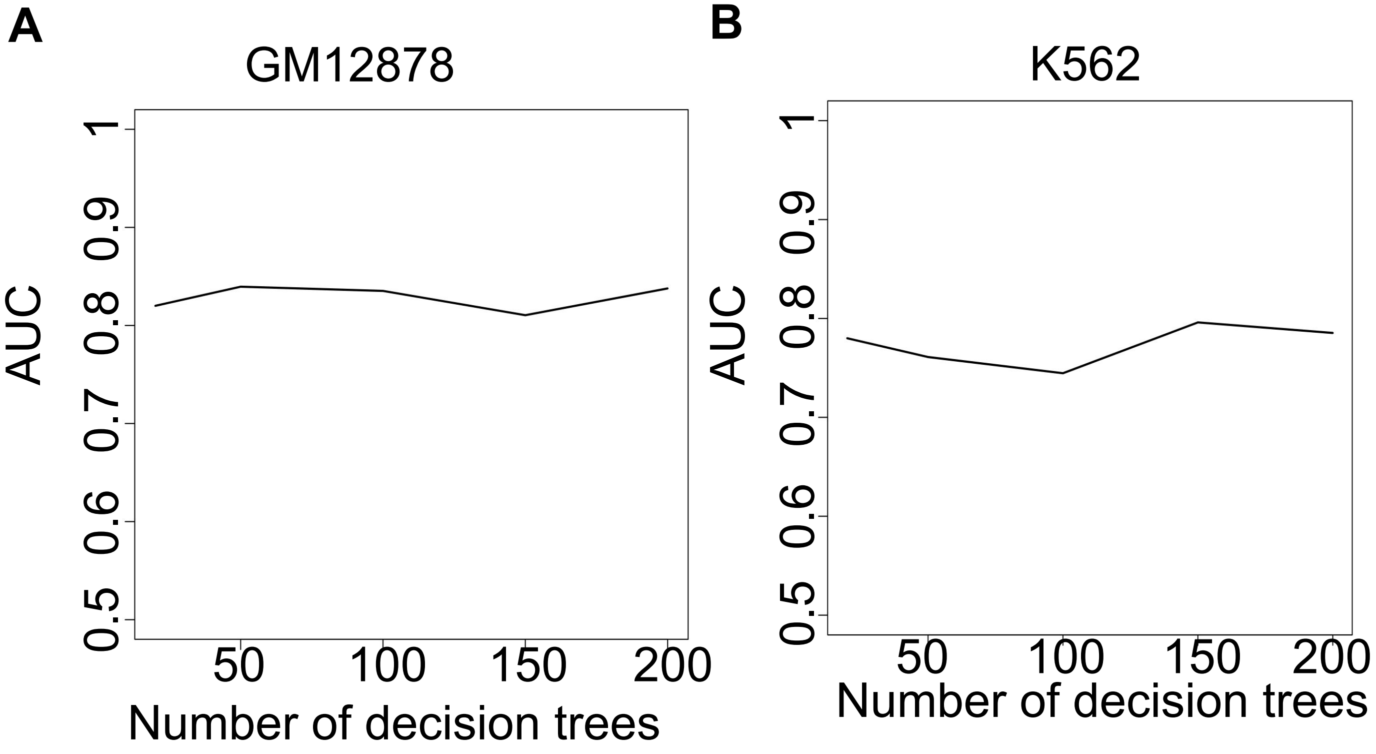

Supplementary Figure 7: Model performance (y-axis) as a function of the number of decision trees (x-axis) used in the random forest model. The AUCs are calculated based on the same data and the same cross-validation procedure. The averaged AUC of cross-validations is shown. Robust performances are observed in (**A**) GM12878 and (**B**) K562.

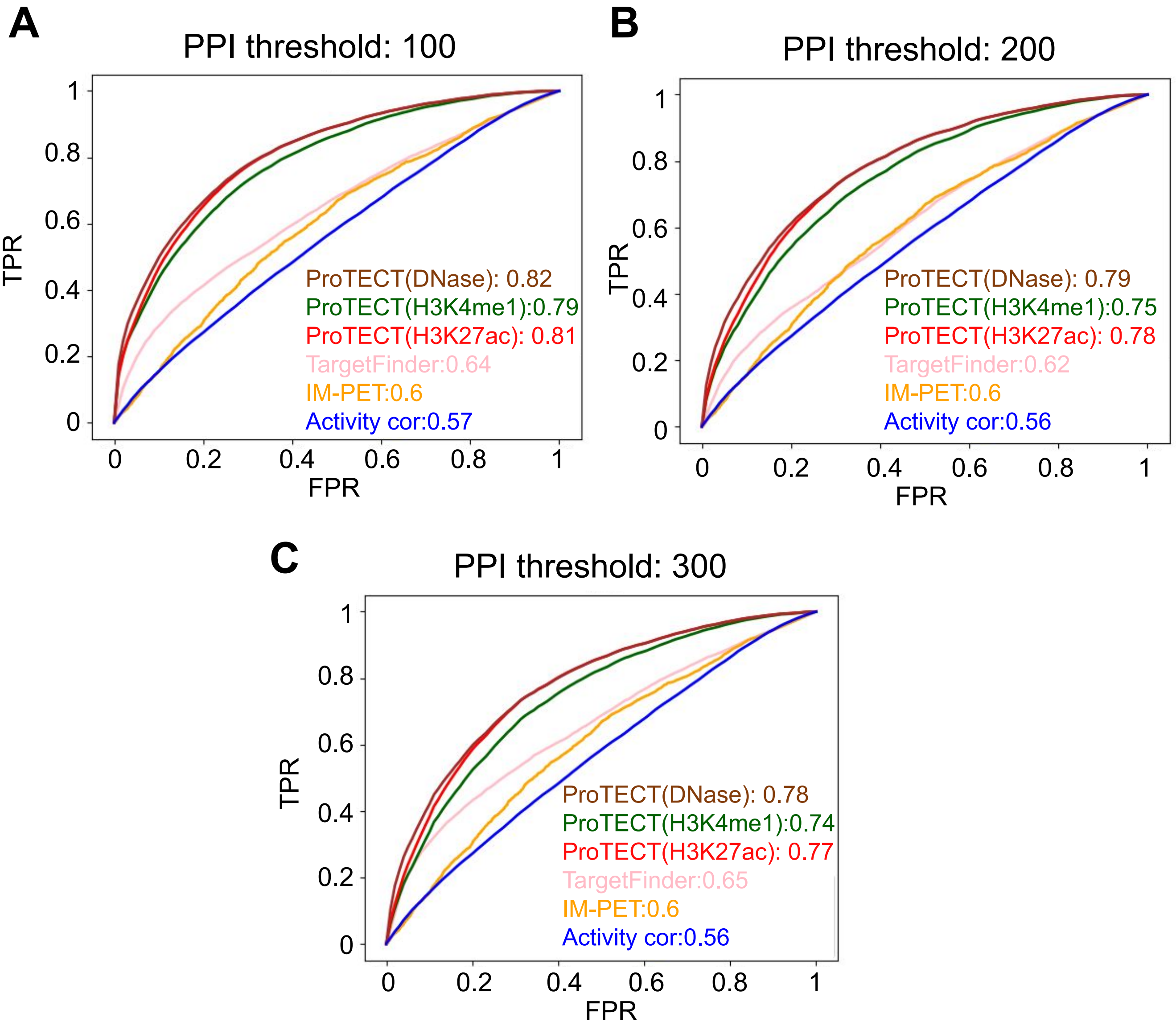

Supplementary Figure 8: Performance of ProTECT using different epigenetic signals for enhancers and thresholds for the PPI confidence scores in GM12878. The enhancer activity is quantified by DNase-seq (brown), H3K4me1 (green) and H3K27ac (red), respectively. The threshold of PPI confidence scores is set to (**A**) 100, (**B**) 200 and (**C**) 300. Only PPIs with 'Experimental' confidence scores greater than the thresholds are used as TF-related features in ProTECT and TargetFinder. In K562. ProTECT achieves AUC=0.8, 0.78 and 0.74 with threshold 100, 200 and 300 respectively. As comparisons, TargetFinder achieves AUC=0.71, 0.69 and 0.69.

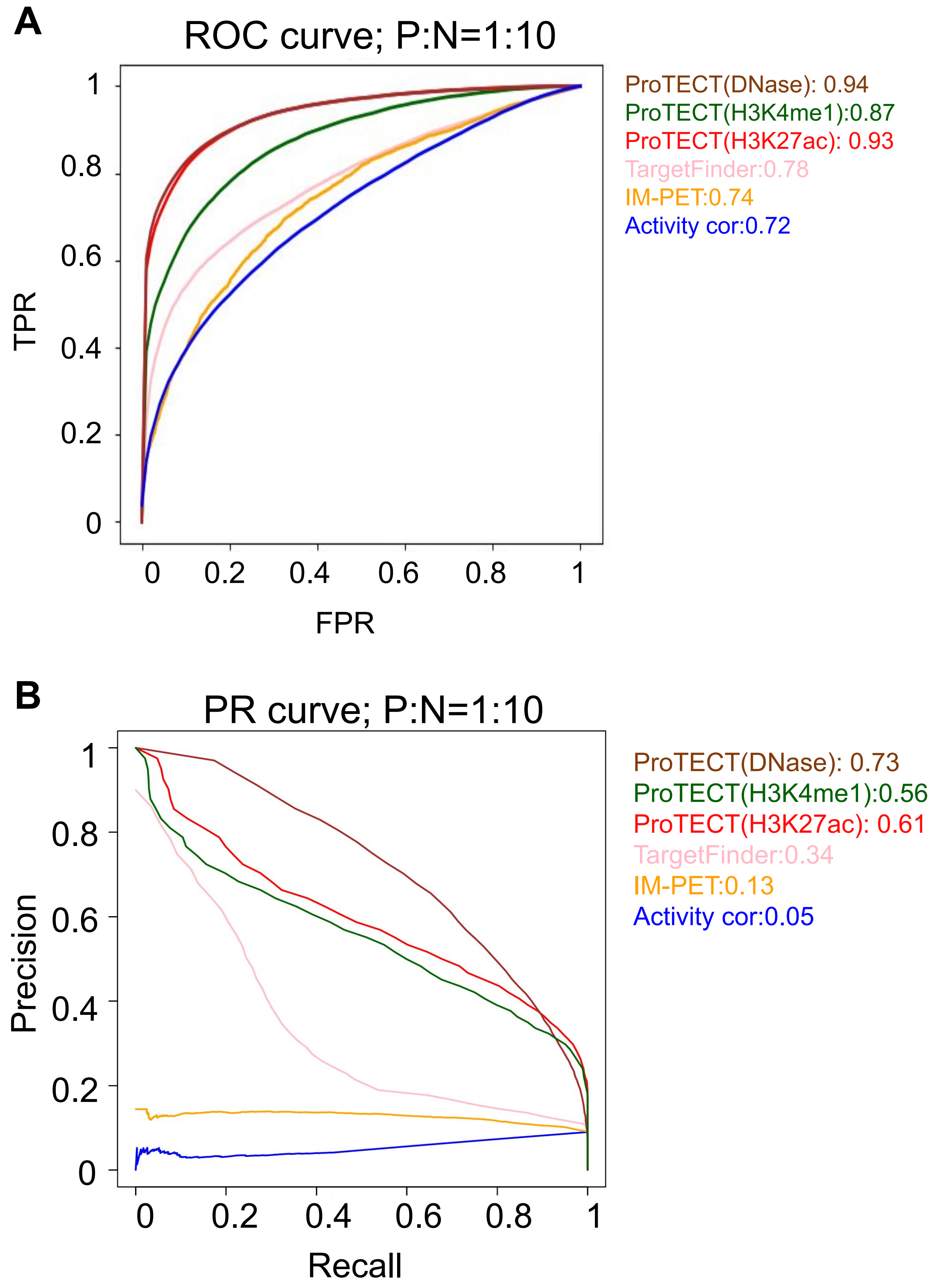

Supplementary Figure 9: Performance comparison based on imbalanced training data, using the genomic bin-split cross-validation procedure. The positive to negative ratio is set to 0.1. The model performance is quantified by (**A**) ROC curves and (**B**) PR curves.

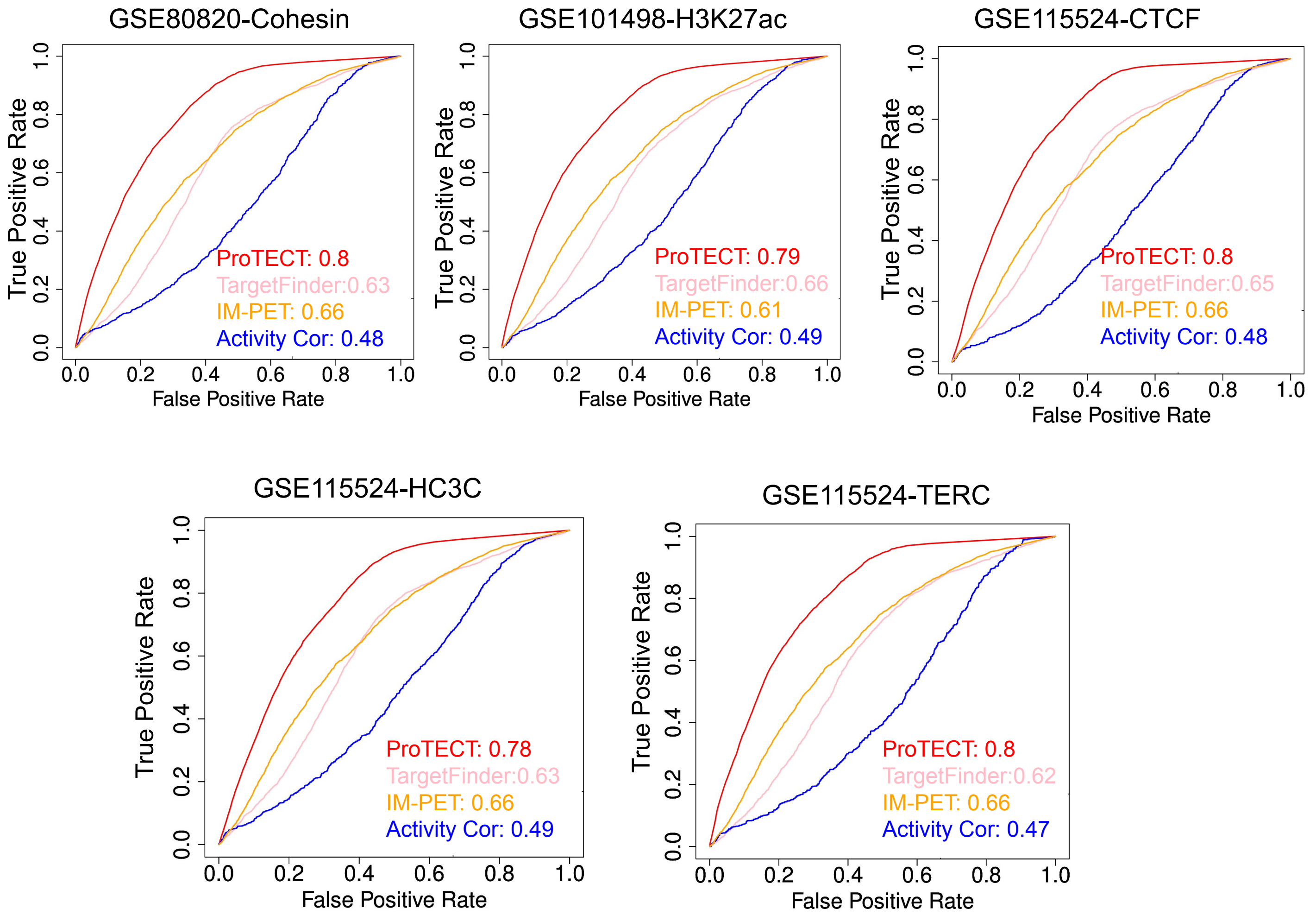

Supplementary Figure 10: Performance comparison using five different Hi-ChIP datasets as the gold-standards in GM12878. The model is trained on the balanced training data. ProTECT uses the DNase-seq signals to quantify enhancer activity. The threshold for the PPI confidence scores is set to 100.

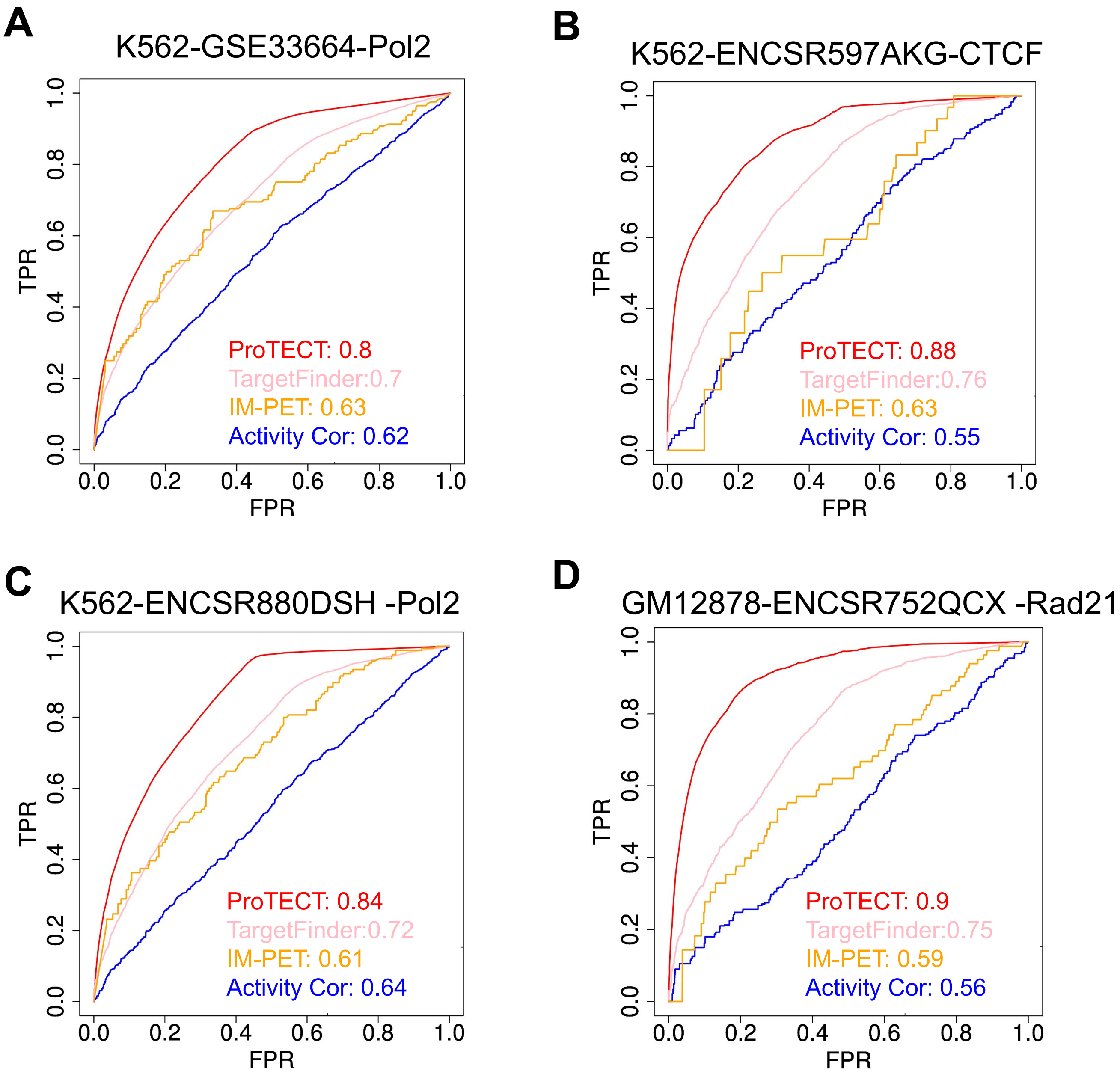

Supplementary Figure 11: Performance comparison using four different ChIA-PET datasets as the gold-standards in (**A**-**C**) K562 and (**D**) GM12878. ProTECT uses the DNase-seq signals to quantify enhancer activities. The threshold for the PPI confidence scores is set to 100.

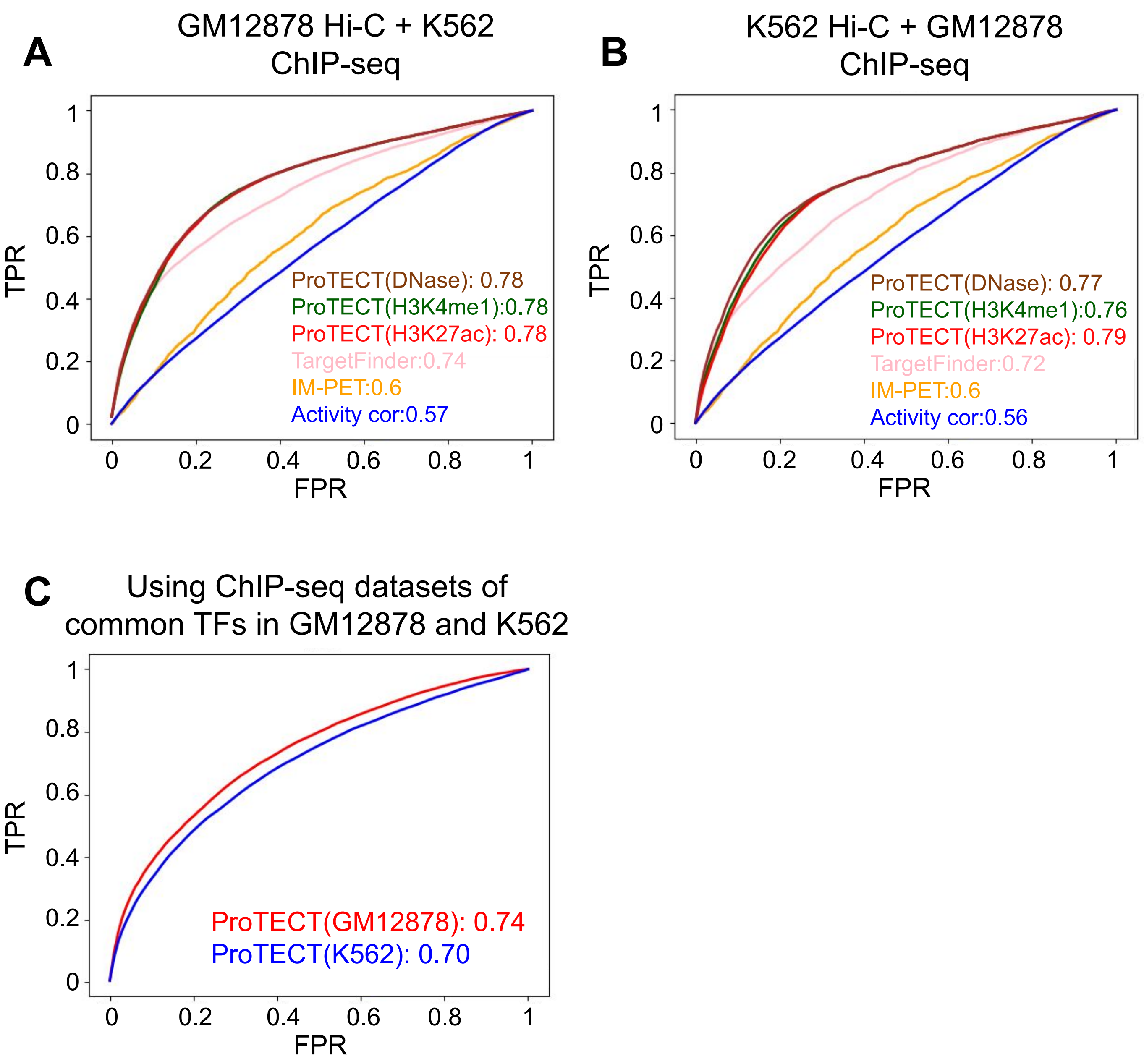

Supplementary Figure 12: Performance comparison based on different combinations of Hi-C data and TF ChIP-seq data. (**A-B**) Performance comparison by pairing Hi-C data and TF ChIP-seq data from different cell-types. (**A**) The training enhancer-gene pairs are generated using the Hi-C data in GM12878. The TF-related features are generated using the TF ChIP-seq datasets in K562. (**B**) The training enhancer-gene pairs are generated using the Hi-C data in K562. The TF-related features are generated using the TF ChIP-seq datasets in GM12878. (**C**) The training data are generated using the Hi-C in GM12878 (red) and K562 (blue) respectively. Only 83 TFs with ChIP-seq data available in both GM12878 and K562 (intersection subset) are used to generate the PPI features.

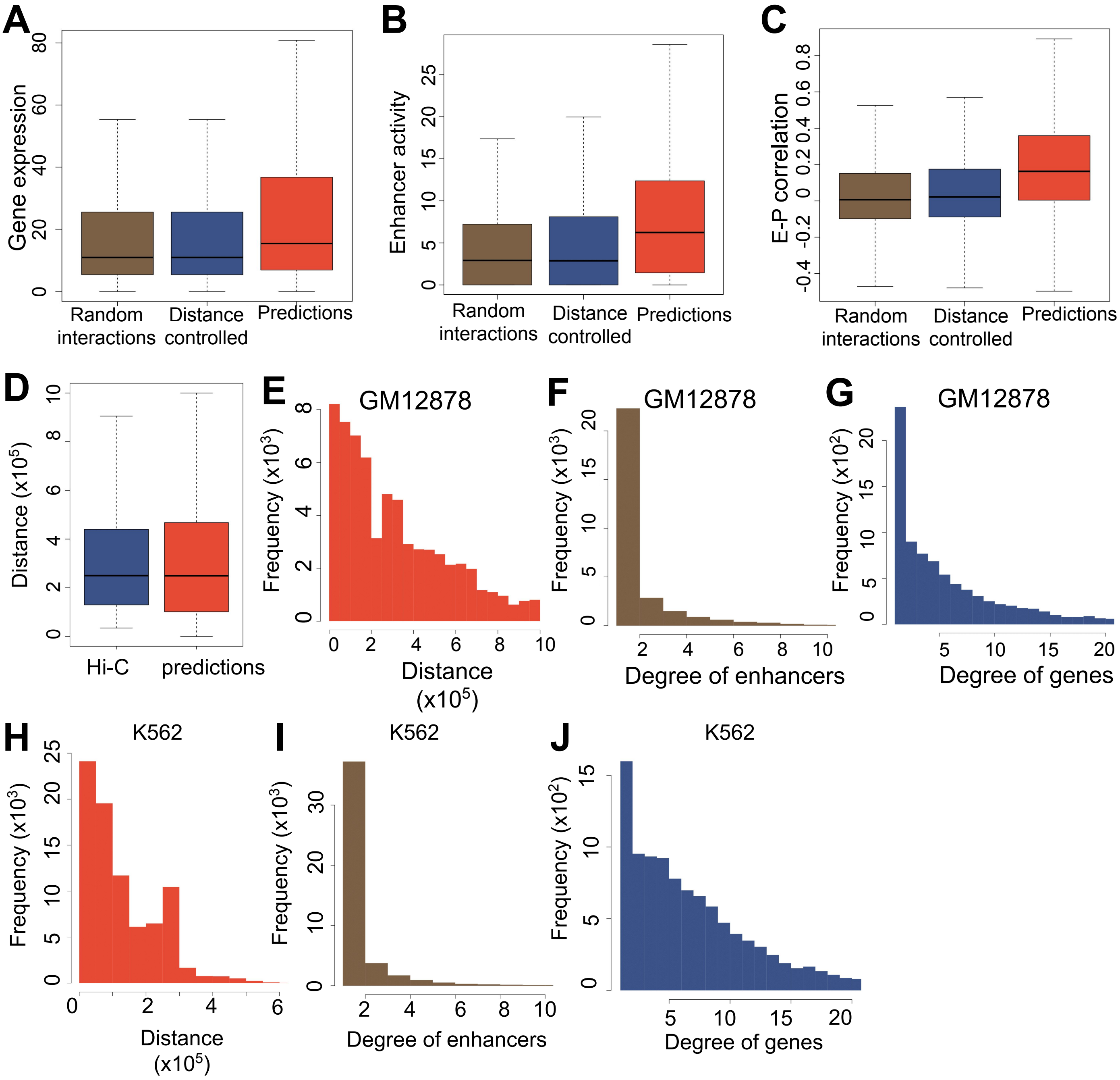

Supplementary Figure 13: Summary of genome-wide predictions by ProTECT in GM12878 and K562. (**A-C**) Compared with controls, the ProTECT predictions have (**A**) higher gene expressions, (**B**) higher enhancer activities, (**C**) higher activity-correlations between enhancers and genes. The controls are generated in the same way as described in Supplementary Figure 1. (**D-E**) Summary of distance distributions of ProTECT predictions in GM12878. (**D**) ProTECT predictions have similar distance distributions as real GM12878 Hi-C interactions. (**E**) The distribution of the genomic distances for predicted enhancer-promoter interactions by ProTECT. (**F-G**) The degree distributions for (**F**) enhancers (i.e. the number of target genes regulated per enhancer), and (**G**) genes (i.e. the number of enhancers co-regulating the same genes), based on ProTECT predictions in GM12878. (**H-J**) The distribution of genomic distances and the degree distributions for enhancers and genes in K562.

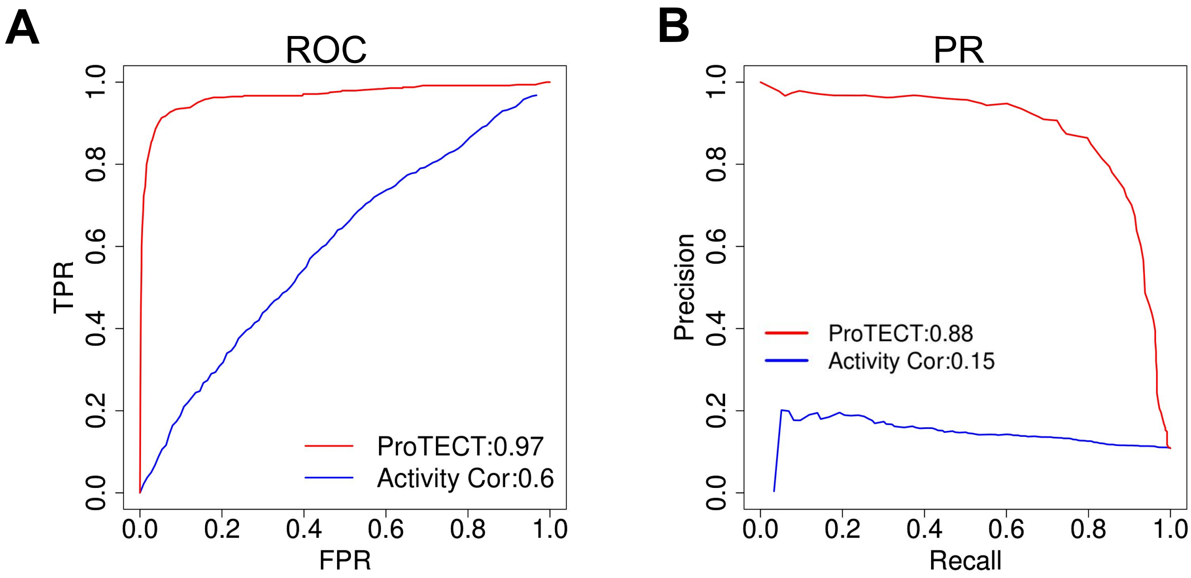

Supplementary Figure 14: Validation of predicted enhancer-gene links with enhancer degrees greater than one. ProTECT is applied to the whole genome to predict enhancer-gene links. The enhancer-gene links are considered as positive samples if the links are supported by the Hi-C interactions and the enhancer has degree greater than one across all positive samples. The negative samples are selected by pairing the same enhancer set as the positive samples with random genes. The positive to negative ratio is set to 0.1. The performance is evaluated by (**A**) ROC curves and (**B**) PR curves.

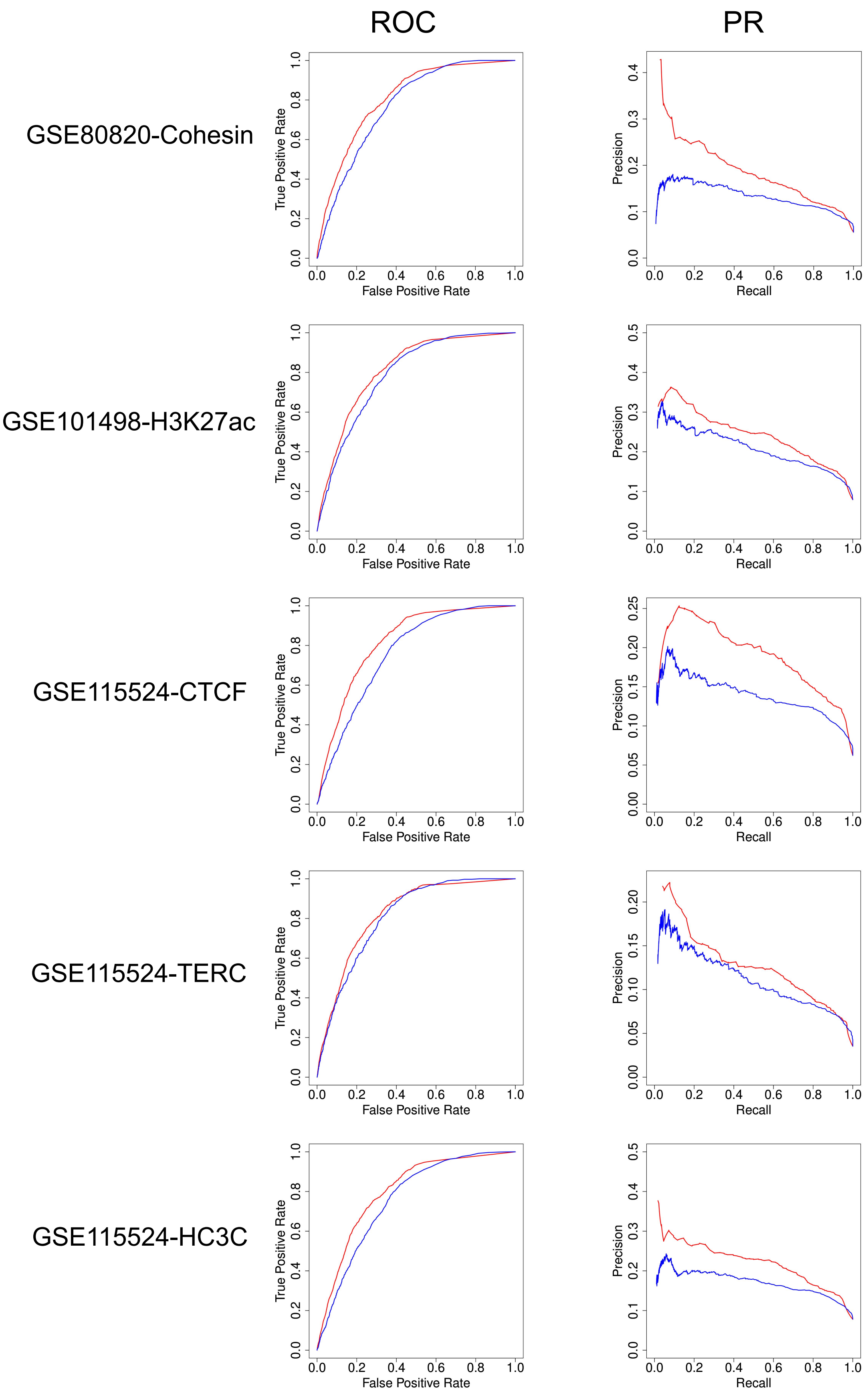

Supplementary Figure 15: Performance comparison with the ABC model in the whole genome. The same set of enhancer-gene pairs are ranked by ProTECT (red) and ABC model (blue) respectively. Five different Hi-ChIP datasets are used as the gold-standards. The performance is evaluated by ROC curves and PR curves.

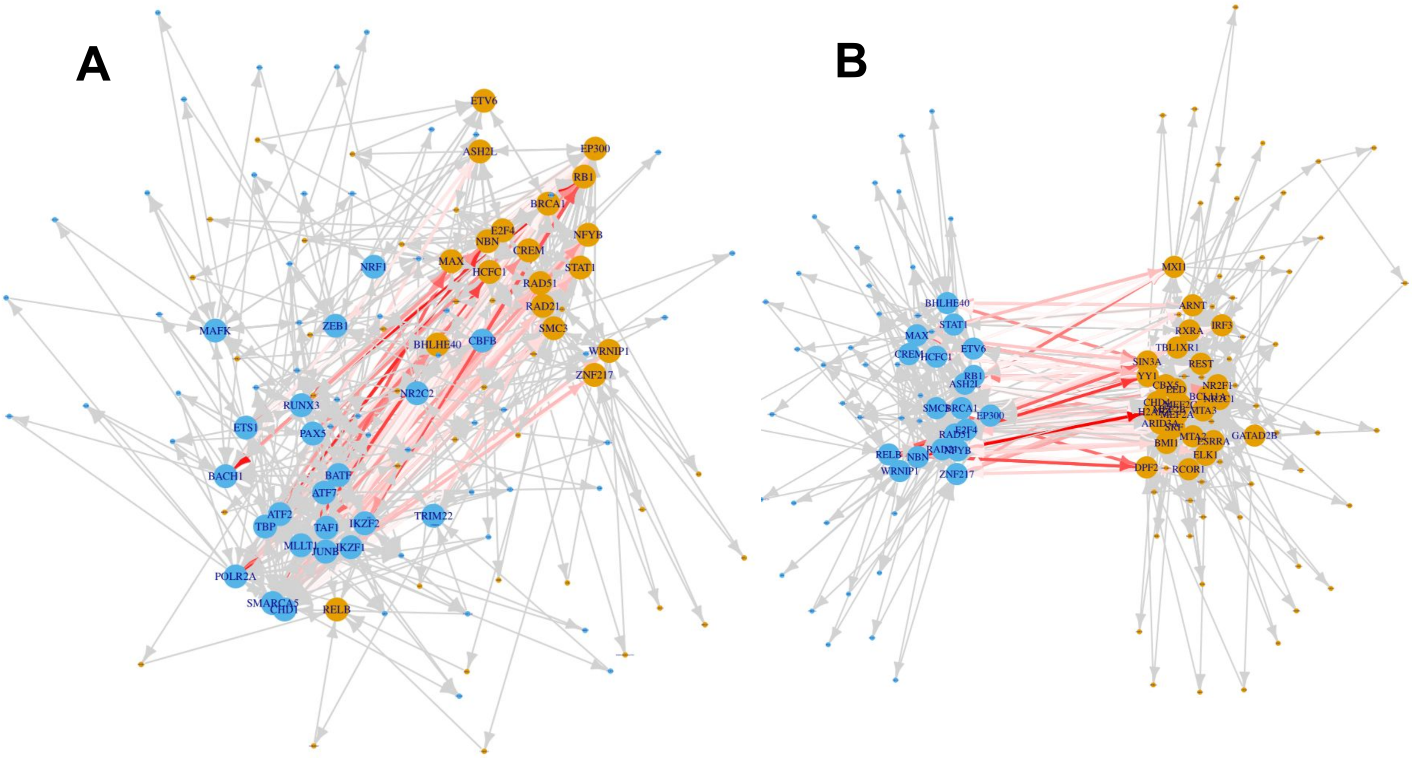

Supplementary Figure 16: Examples of prioritized module-level TF PPI features. The two module-level TF PPI features are selected based on the highest feature importance inferred by the random forest model. Two types of nodes are included in the network: TFs (large size) and non-TF proteins (small). Nodes from the first module are colored as blue and TFs from the second module are colored as orange. Edges represent the inter-module TF-level PPIs that connect the TF module pairs. Edges point from enhancer-binding TFs to promoter-binding TFs. The edges are colored by the occurrence frequencies of TF PPIs in ProTECT predictions. Important TF-level PPIs in each module-level feature are further prioritized based on the occurrence frequencies, e.g.RELB-YY1 and SMC3-POLR2A.

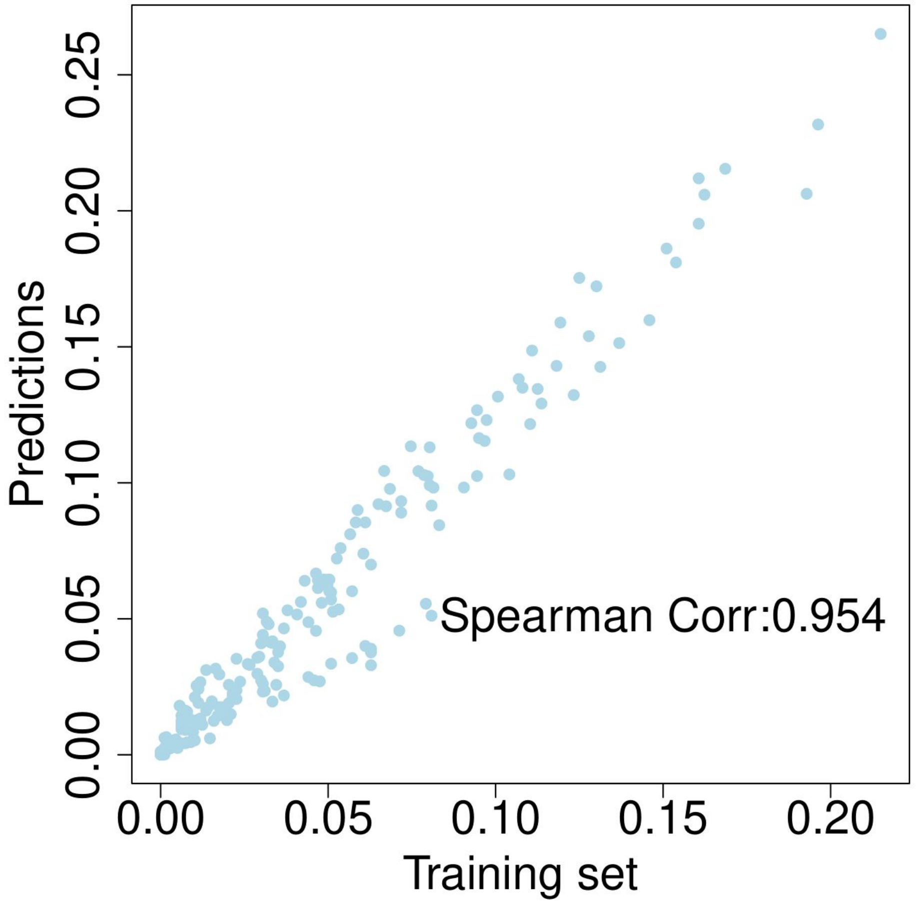

Supplementary Figure 17: Comparing the TF-level PPI abundance scores in the Hi-C supported enhancer-gene links (training set, x-axis) and the ProTECT predictions (y-axis). Dots represent the TF PPIs. The Spearman correlation is 0.954.

A Un-directional TF PPI features

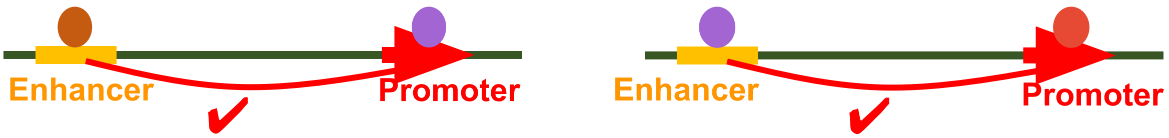

	TF-TF abundance score	Fold
ATF2-SMARCA5	0.136269	1.39
SMARCA5-ATF2	0.194012	
IKZF1-CREM	0.110332	1.17
CREM-IKZF1	0.0941	

B Directional TF PPI features

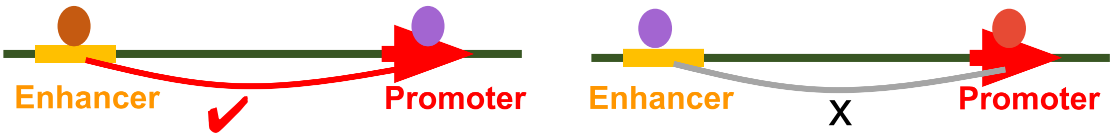

	TF-TF abundance score	Fold
EP300-POL2R2A	0.193	9.19
POL2R2A-EP300	0.021	
SMC3-MXI1	0.234	7.8
MXI1-SMC3	0.030	

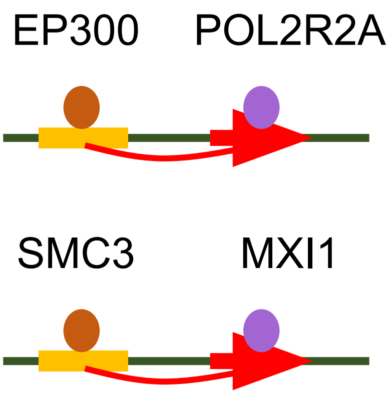

Supplementary Figure 18: Identification of the directions of TF PPI features. For each pair of TF PPI features with opposite directions, the fractions of predicted enhancer-promoter interactions containing the specific TF PPI features are used as the abundance scores. (**A**) Examples of un-directional TF PPI features, where the abundance scores of two directional features are similar to each other. (**B**) Examples of directional TF PPI features, where the abundance scores of the two directional features are substantially different.

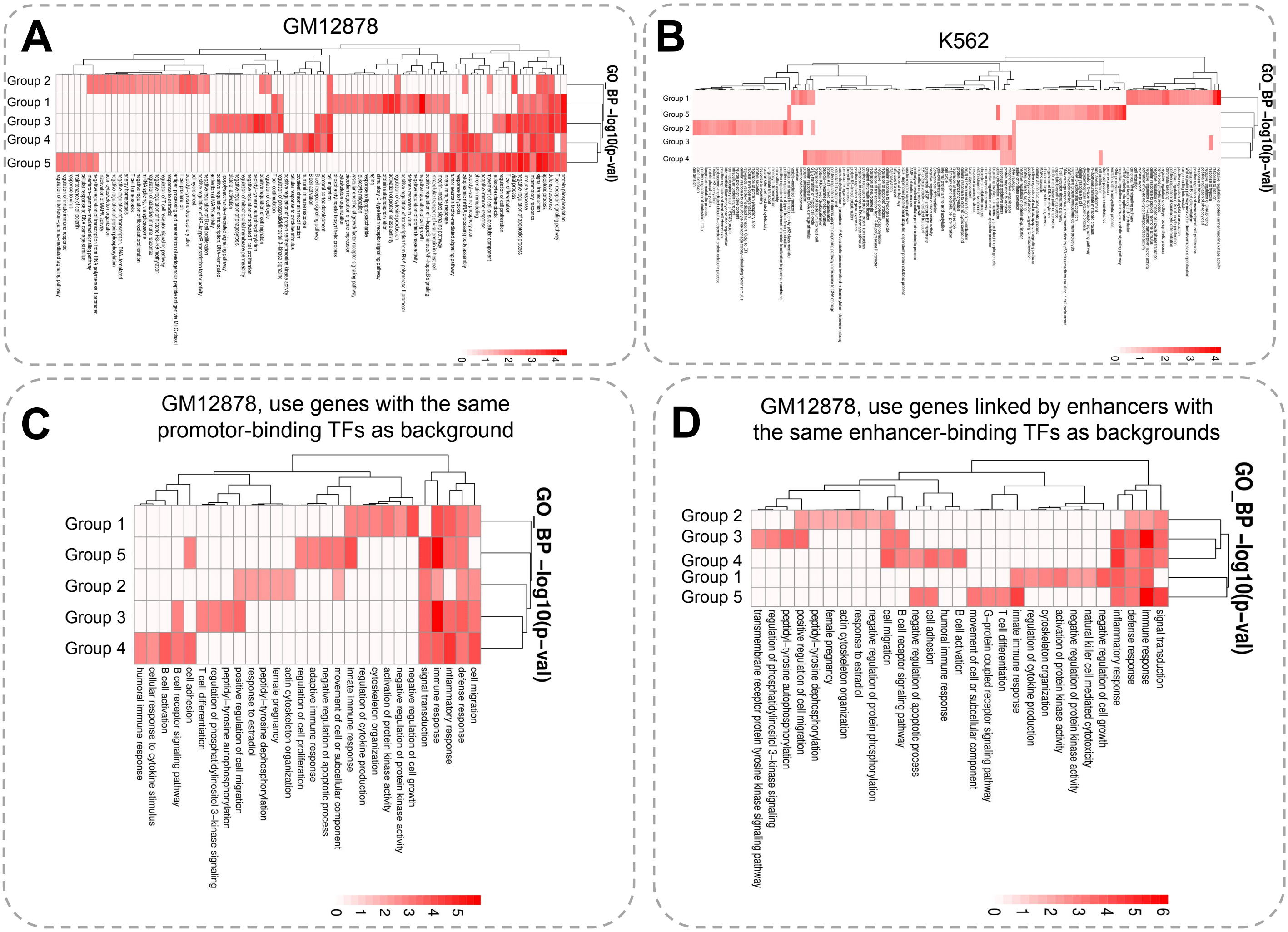

Supplementary Figure 19: Differential pathway enrichments of genes regulated by different module-level TF PPIs based on the ProTECT predictions. (**A**-**B**) Using all genes as the background gene sets in (**A**) GM12878 and (**B**) K562. (**C**) The genes with the same promoter-binding TFs are used as the background gene sets in GM12878 for the GO analysis. (**D**) The genes linked by the enhancers with the same enhancer-binding TFs are used as the background gene sets in GM12878 for the GO analysis.

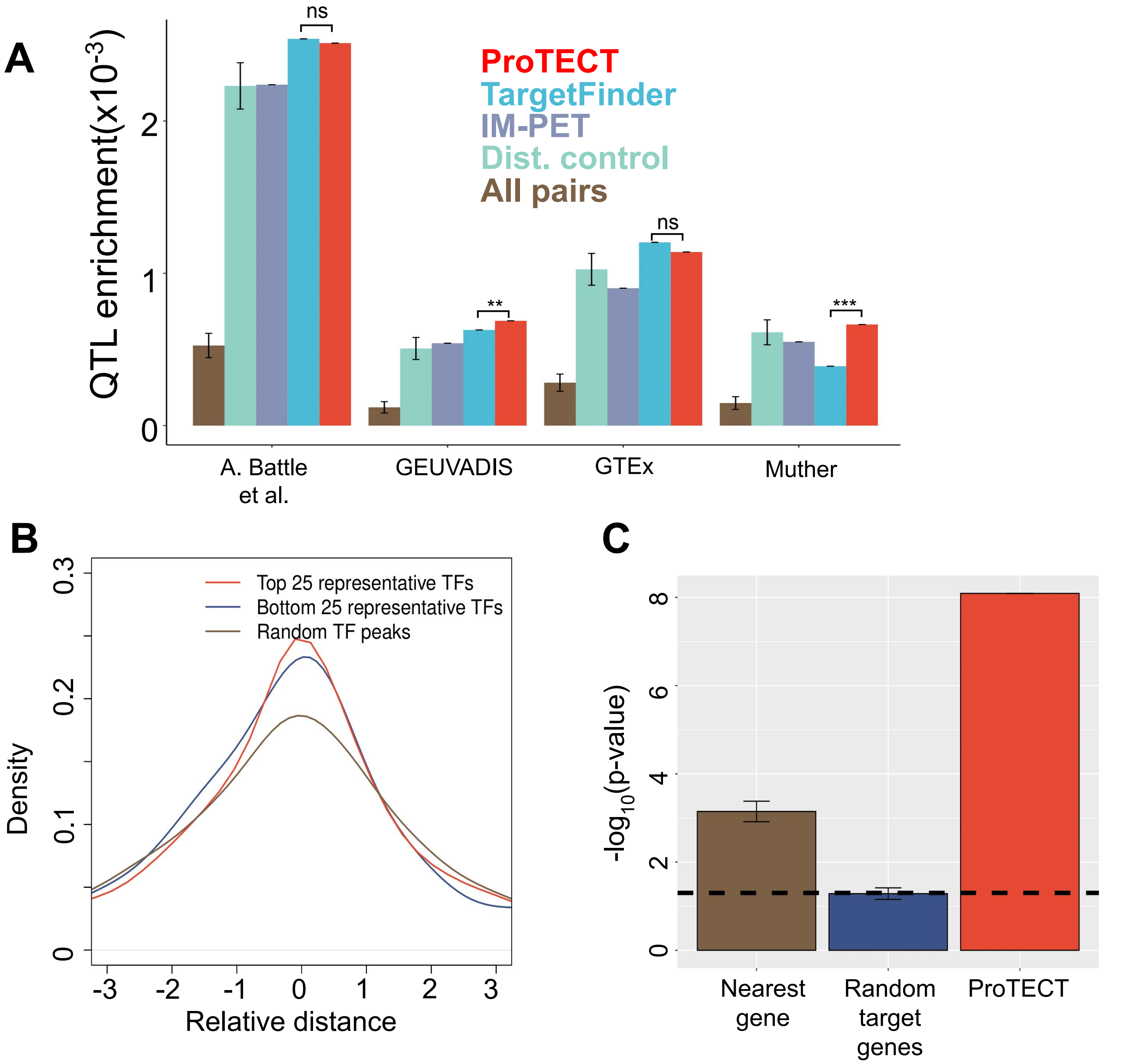

Supplementary Figure 20: QTL enrichment analysis in K562. (**A**) Enrichment of ProTECT predictions with cis-eQTLs (y-axis) from multiple datasets (x-axis). Two versions of controls are generated: 1) Random pairs (brown): randomly pairing enhancers and promoters within 2Mb distance windows. 2) Distance controlled (blue): randomly pairing enhancers and genes while the genomic distances follow the same distribution as ProTECT predictions (red). Controls are generated 1,000 times and standard deviations are used for error bars. Enrichments of TargetFinder and IM-PET are also included. Empirical p-values are calculated. (***: p-value < 10^{-3}, **: p-value < 10^{-2}) (**B**) Relative genomic distance distribution between cis-eQTL SNPs and the summits of ChIP-seq peaks of the prioritized TFs by the model (red). The relative distance is calculated as the distance between cis-eQTL SNPs and TF ChIP-seq peak summits, normalized by the size of TF peaks. The same number of peaks of bottom-ranked TFs (grey) and randomly selected enhancer-binding TFs (blue) are used as controls. The p-value equals to 0.0518 based on the Kolmogorov-Smirnov test. (**C**) Trans-eQTL analysis in K562. The hypergeometric test is used to test the enrichment of overlaps between enhancer-mediated TF-gene pairs and trans-eQTLs, whose SNPs located in the TF's gene body and the eQTL target gene is the same as the TF's target gene (red). The $-\log_{10}$(p-value) of the hypergeometric test is shown. The p-values are highly significant in ProTECT predictions (p-value=8.12×10^{-9}) compared with two controls: 1) nearest gene to enhancers (p-value=0.052, brown), and 2) random target genes (p-value=7.13×10^{-4}, blue).

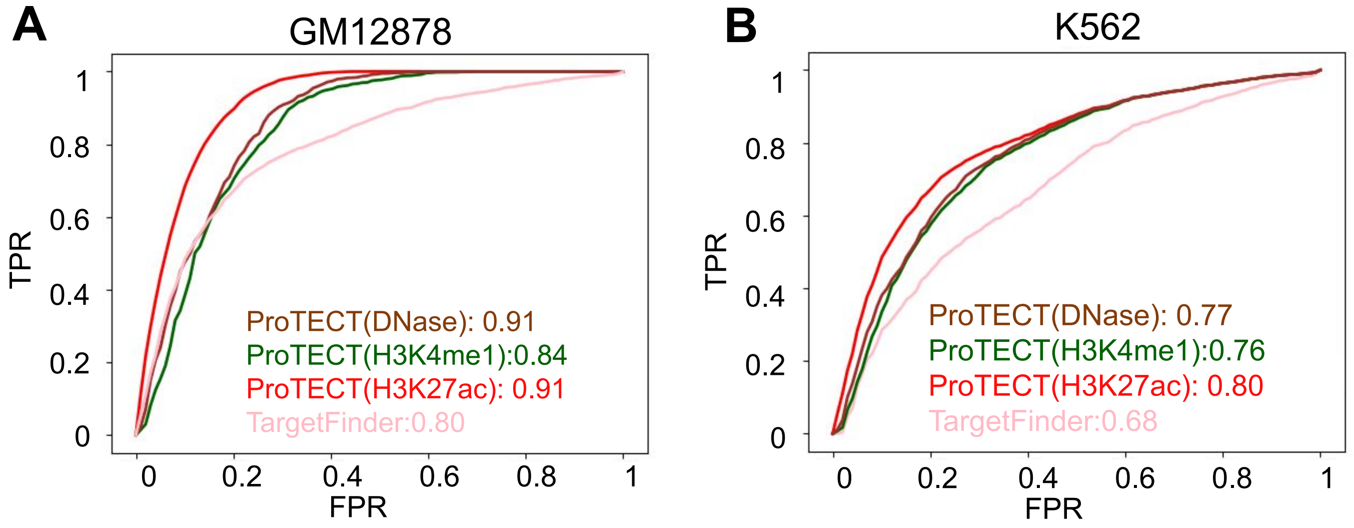

Supplementary Figure 21: ProTECT predicts enhancer-gene links based on the imputed TF binding sites. The TF binding sites are imputed by overlapping TF motifs with cell-type specific DNase-seq peaks. For each TF, strong motifs within DNase-seq peaks across the whole genome are considered as the imputed TF binding sites. ProTECT uses the imputed TF binding sites to generate the TF PPI features. (**A**) In GM12878, TF binding sites for 128 TFs are imputed and used. (**B**) In K562, TF binding sites for 270 TFs are imputed and used.

Supplementary methods

The supplementary methods contain three sections:

- Detailed pre-processing steps for input datasets
- Notes on model parameter settings
- Practical issues of algorithm implementation and downstream analyses

Detailed pre-processing steps for input datasets

The significant chromatin interactions can be collected from datasets of Hi-C (1), ChIA-PET (2) or Capture Hi-C (3). In this paper, we used the high-resolution cell-type specific Hi-C interactions in GM12878 and K562 from the ENCODE project (GSE63525) (1). The topological associated domains (TADs) can be mapped using the computational models, e.g. Arrowhead (1), on Hi-C chromatin contact maps. In this paper, we collected the contact domain annotations in GM12878 and K562 from GEO (GSE63525) (1).

Enhancer coordinates are based on ENCODE and Roadmap Epigenomics enhancer annotations for GM12878 and K562 cell-lines (4,5). We quantify the cell-type-specific enhancer activities using DNase epigenomic signals (5). Specifically, the enhancer activity for enhancer i in cell type j is defined as $E_{ij} = Ave\_signal^{DNase}_{Cell_{type_j}}(bins\ overlapping\ with\ enhancer\ i)$. The activities of 697,876 enhancers across 56 cell-types are summarized into an enhancer activity matrix.

Promoters of genes are defined as +/-1kb around transcriptional start sites (TSS), based on gene annotations from GENCODE v17 (6). Cell-type-specific gene expressions are quantified by the averaged RPKM signals of each gene. Based on the Roadmap Epigenomics RNA-seq datasets across different cell-types and tissues, potential housekeeping genes are removed from the analysis if the gene expression variances are ranked as the lowest 5% among all genes.

The activity correlation coefficients are calculated for each enhancer-promoter interaction based on the enhancer activity profiles and the gene expression profiles across 56 cell-types. Both enhancer activity matrix and gene expression matrix are quantile normalized to remove the batch effects across different cell-types or tissues.

The narrow peak annotations of TF ChIP-seq experiments called by MACS2 (7) can be downloaded from the ENCODE project website. As quality control, we applied three criteria to filter the TF ChIP-seq datasets, in order to select the dataset with the best quality for each specific TF: (1) TF ChIP-seq datasets for treated transcription factors are removed. (2) TF ChIP-seq datasets generated by paired-end experiments are preferred if available. (3) FRiP (Fraction of Reads in Peaks) scores are calculated for each TF ChIP-seq replicate. For each TF ChIP-

seq experiment, the averaged FRiP score across all replicates is used to quantify the data quality. Based on these three criteria, the TF ChIP-seq dataset with the highest averaged FRiP score is selected. In total, we selected 129 TF ChIP-seq datasets in GM12878 and 270 TF ChIP-seq datasets in K562.

The GTEx cis-eQTLs are collected with version 7 (8) . For the cis-eQTL enrichment analysis in predicted enhancer-promoter interactions, ~20,000 significant cis-eQTLs are selected based on gene-wise multiple hypothesis correction of p-values, i.e. q-values, from GTEx (eGenes), where only one SNP with the smallest q-value for each gene is considered as significant. This set of cis-eQTLs is highly conservative, thus providing a gold-standard for the enrichment analysis of cis-eQTLs in predicted enhancer-promoter interactions. For the enrichment analysis of eQTL SNPs around TF binding sites within enhancers, the eQTL SNPs are selected based on two criteria: 1) The nominal p-values are smaller than $5x10^{-8}$; 2) The minor allele frequency is greater than 0.05. By applying these two filters, 318,026 eQTL SNPs are selected for the analysis.

Notes on model parameter settings

Hierarchical community detection on the PPI network of transcription factors

The undirected weighted PPI network is constructed based on the PPI data, where nodes represent proteins, edges represent PPIs and the edge weights represent PPI confidence scores in the 'Experiments' category. To resolve the dense connections and reveal the basic topology of the PPI network, we first prune the PPI edges using PPI 'Experiment' confidence scores. Only PPI edges with confidence scores greater than 500 are maintained in the network for module detections. The distance between two nodes in the graph are calculated based on the random walks on the PPI network. As a free parameter, the number of steps p of random walks need to be selected to capture both local and global topological properties of nodes. To select the optimal value of p, we tested a series of values for network community detection based on the random walks, and selected the one with the highest modularity score ($p = 20$) (Supplementary Figure 4A). A hierarchical tree is then constructed to identify the hierarchical PPI modules of TFs. By applying the bottom-up merging strategy on the tree, a two-layer hierarchical structure of TF modules are identified, *i.e.* small size S-modules with the maximum size of $S_{max}$and large size L-modules with the maximum size of L_{max}. To set the optimal number of S_{max} and L_{max}, we tested a series of values maximum module sizes in hierarchical clustering and calculated the corresponding modularity scores. The S_{max} is set to be the value corresponding to the highest modularity score, while the $L_{max}$is set to be the value corresponding to the elbow point of the modularity curves. Therefore, the S_{max} represents the best module assignment based on the modularity score and the L_{max} balances the module size and the modularity score. Consequently, we identified 401 S-modules and 234 L-modules

in GM12878. Similarly, 526 S-modules and 313 L-modules are identified in K562 (Supplementary Figure 4C-D).

Random forest model training

We used the Random Forest model with 50 decision trees to predict cell-type specific enhancer-promoter interactions based on the generated features. The AUC based on the cross-validation is used to quantify the model performance. We tested the Random Forest models with different numbers of trees and observed a flat AUC curve, which suggests that the model is robust to the number of trees (Supplementary Figure 7).

Practical issues of algorithm implementation and downstream analysis

Genome-wide application to predict long-range enhancer-promoter interactions

To predict significant enhancer-promoter interactions, we generated the high dimensional features for all potential enhancer-promoter interactions. Considering the huge amounts of potential pairs, we only focused on enhancer-promoter pairs with genomic distance smaller than 2Mb. This results in ~18 million potential pairs. The TF-level PPI features were transformed into module-level TF PPI features based on the same strategy learnt from the model training, and the generated feature matrix was then used for genome-wide predictions.

trans-eQTL enrichment analysis for enhancer-mediated TF-gene pairs

For ProTECT predictions of enhancer-promoter interactions, the prioritized enhancer-mediated TF-gene pairs are identified using the 75% top-ranking enhancer-promoter interactions (based on q-values). As the genomic background, a whole pool of potential TF-gene pairs are generated by considering all TF binding sites in enhancers and predicted enhancer-promoter interactions. The trans-eQTLs are then used to validate the prioritized TF-gene pairs from ProTECT. A TF-gene pair is considered to be supported by trans-eQTLs from the Pancan database (9) based on two criteria: 1) the SNP of a trans-eQTL is located in the extended TF's gene body (including -10kb from TSS); 2) The TF-gene pair share the same target gene as the SNP of trans-eQTL. Among the genomic background of all potential TF-gene pairs, 9 TF-gene pairs are supported by trans-eQTLs, while, strikingly, 6 out of the 9 TF-gene pairs are prioritized by ProTECT. Hypergeometric tests were used to evaluate the statistical significance on the overlaps.

References

1. Rao, S.S., Huntley, M.H., Durand, N.C., Stamenova, E.K., Bochkov, I.D., Robinson, J.T., Sanborn, A.L., Machol, I., Omer, A.D., Lander, E.S. *et al.* (2014) A 3D map of the human genome at kilobase resolution reveals principles of chromatin looping. *Cell*, **159**, 1665-1680.
2. Roy, S., Siahpirani, A.F., Chasman, D., Knaack, S., Ay, F., Stewart, R., Wilson, M. and Sridharan, R. (2015) A predictive modeling approach for cell line-specific long-range regulatory interactions. *Nucleic Acids Res*, **43**, 8694-8712.
3. Jung, I., Schmitt, A., Diao, Y., Lee, A.J., Liu, T., Yang, D., Tan, C., Eom, J., Chan, M., Chee, S. *et al.* (2019) A compendium of promoter-centered long-range chromatin interactions in the human genome. *Nat Genet*, **51**, 1442-1449.
4. Consortium, E.P. (2012) An integrated encyclopedia of DNA elements in the human genome. *Nature*, **489**, 57-74.
5. Roadmap Epigenomics, C., Kundaje, A., Meuleman, W., Ernst, J., Bilenky, M., Yen, A., Heravi-Moussavi, A., Kheradpour, P., Zhang, Z., Wang, J. *et al.* (2015) Integrative analysis of 111 reference human epigenomes. *Nature*, **518**, 317-330.
6. Harrow, J., Denoeud, F., Frankish, A., Reymond, A., Chen, C.K., Chrast, J., Lagarde, J., Gilbert, J.G., Storey, R., Swarbreck, D. *et al.* (2006) GENCODE: producing a reference annotation for ENCODE. *Genome Biol*, **7 Suppl 1**, S4 1-9.
7. Zhang, Y., Liu, T., Meyer, C.A., Eeckhoute, J., Johnson, D.S., Bernstein, B.E., Nusbaum, C., Myers, R.M., Brown, M., Li, W. *et al.* (2008) Model-based analysis of ChIP-Seq (MACS). *Genome Biol*, **9**, R137.
8. Consortium, G.T., Laboratory, D.A., Coordinating Center -Analysis Working, G., Statistical Methods groups-Analysis Working, G., Enhancing, G.g., Fund, N.I.H.C., Nih/Nci, Nih/Nhgri, Nih/Nimh, Nih/Nida *et al.* (2017) Genetic effects on gene expression across human tissues. *Nature*, **550**, 204-213.
9. Gong, J., Mei, S., Liu, C., Xiang, Y., Ye, Y., Zhang, Z., Feng, J., Liu, R., Diao, L., Guo, A.Y. *et al.* (2018) PancanQTL: systematic identification of cis-eQTLs and trans-eQTLs in 33 cancer types. *Nucleic Acids Res*, **46**, D971-D976.
