# Supplementary material for: Predict long-range enhancer regulation based on protein–protein interactions between transcription factors
Source: Nucleic Acids Res. 2021 Sep 27;49(18):10347–68. doi: 10.1093/nar/gkab841 (PMC8501976; doi:10.1093/nar/gkab841)
Supplement: gkab841_Supplemental_Files [file gkab841_supplemental_files.zip › Supplementary_Table_Legends.docx]

**Supplementary Table Legends**

**Supplementary Table 1**: Summary of sources and accession numbers for datasets used in the paper. Three pages are included: 1) Accession numbers of all TF ChIP-seq datasets for GM12878 and K562; 2) Sources of DNase-seq, RNA-seq, enhancer annotation, gene annotation and TF motif annotations; 3) Accession numbers of chromatin interaction datasets, including Hi-C, ChIA-PET and Hi-ChIP.

**Supplementary Table 2**: List of transcription factors belong to the NR, bHLH, and bZIP families, whose intratypic dimerizations are removed from the PPI network. Each column corresponds to one specific family.
